# Deep learning framework for hourly air pollutants forecasting using encoding cyclical features across multiple monitoring sites in Beijing

**DOI:** 10.1038/s41598-025-05472-5

**Published:** 2025-07-01

**Authors:** Abdel Salam Alsabagh, Omer A. Alawi, Haslinda Mohamed Kamar, Ahmed Adil Nafea, Mohammed M. AL-Ani, Hussein A. Mohammed, S. N. Kazi, Atheer Y. Oudah, Zaher Mundher Yaseen

**Affiliations:** 1 Department of Mechanical Engineering, Faculty of Engineering Technology, Al-Balqa Applied University, Amman, Jordan; 2 Department of Thermofluids, Faculty of Mechanical Engineering, Universiti Teknologi Malaysia, 81310 Skudai, Johor Bahru, Malaysia; 3 Department of Power Mechanics Engineering Techniques, Engineering Technical College, Al-Bayan University, Baghdad, 10011 Iraq; 4 Department of Artificial Intelligence, College of Computer Science and IT, University of Anbar, Ramadi, Iraq; 5 Center for Artificial Intelligence Technology (CAIT), Faculty of Information Science and Technology, Universiti Kebangsaan Malaysia (UKM), Bangi, Selangor Malaysia; 6 Mechanical Engineering Department, King Fahd University of Petroleum and Minerals (KFUPM), Dhahran, 31261 Saudi Arabia; 7 Department of Mechanical Engineering, Faculty of Engineering, Universiti Malaya, 50603 Kuala Lumpur, Malaysia; 8 Department of Computer Sciences, College of Education for Pure Science, University of Thi-Qar, Nasiriyah, 64001 Iraq; 9 Information and Communication Technology Research Group, Scientific Research Center, Al-Ayen University, Nasiriyah, 64001 Iraq; 10 Civil and Environmental Engineering Department, King Fahd University of Petroleum and Minerals, 31261 Dhahran, Saudi Arabia

**Keywords:** Air quality forecasting, Air pollution monitoring, Deep learning, Early warning systems, Environmental assessment, Environmental impact, Environmental impact, Computational science, Scientific data

## Abstract

Environmental managers and citizens alike are concerned with air quality. Early warning systems for air pollution are essential to prevent health issues and implement effective prevention strategies. This paper proposes a comprehensive, reliable system with air quality prediction and assessment modules for China’s air pollution. In this study, six air pollutants were observed, including Carbon Monoxide (CO), Nitrogen Dioxide (NO_2_), Ozone (O_3_), Sulphur Dioxide (SO_2_), Fine particulate matter (PM_2.5_), and Coarse particulate matter (PM_10_). The current dataset includes hourly air pollutants data from 10 national air-quality monitoring sites, such as Aotizhongxin, Changping, Dongsi, Guanyuan, Huairou, Nongzhanguan, Shunyi, Tiantan, Wanliu, and Wanshouxigong. The dataset was recorded hourly from 01/03/2013 to 28/02/2017. Deep Neural Networks (DNNs) and Convolutional Neural Networks (CNNs) were developed with both unencoded and encoded features to address the forecasting challenge of multivariate time series, specifically in predicting air pollution concentrations. The results showed that, the top accuracy was as follows: 93.8% at the Wanshouxigong station using CNN-Encoded, 91.9% at the Nongzhanguan station using (DNN-Encoded and CNN-Encoded), 93.4% at Aotizhongxin station using DNN-Encoded, 96.2% at Nongzhanguan station using DNN-Encoded, 94% at Dongsi station using CNN-Unencoded, and 92.4% at Aotizhongxin station using (CNN-Unencoded and DNN-Encoded) in forecasting CO, NO_2_, O_3_, PM_2.5_, PM_10_ and SO_2_ pollutants, respectively. The findings indicated that the suggested approaches are efficient and dependable for environmental supervisors in the monitoring and management of air pollution.

## Introduction

### General background of study

Air pollution is a major issue worldwide due to its negative effects on human health, the environment, and the climate^1^. Among several criterion pollutants for determining the levels of air pollution, six parameters are generally considered highly concerning, including SO_2_, NO_2_, CO, O_3_, PM_2.5_, and PM_10_^2^. According to data from the World Health Organisation (WHO), nine out of ten individuals breathe air with high levels of these pollutants, which is beyond the standard limits of the WHO. Air pollution will cause seven million premature deaths globally each year. In addition to impairing vision, air pollution can affect the balance of solar radiation directly or indirectly^3^ and might even spark more severe weather conditions like drought and flooding^4^. The concentration of air pollutants in ambient air can be influenced by meteorological conditions, which can also contribute to the mobility, emission, chemical synthesis, and deposition of these pollutants. This is crucial to any actions or management initiatives to reduce air pollution^5^.

Variations in meteorological data may result in inaccurate conclusions about management effectiveness or intervention. This can make it difficult to determine the trends in different air contaminants accurately^6^. Therefore, it is essential to distinguish weather effects from data trends on air quality and to identify the precise policy-driven changes in air quality^4^. Determining air pollution parameters using a theoretical model based on algorithmic methods was a reliable alternative method. Regression models mathematically represent statistical correlations, quantifying the influence of multiple independent variables on a single dependent variable. Since big data is gradually affecting every aspect of daily life. In the future, data resources will become more and more valuable. The use of data and technology from big data thinking and artificial intelligence (AI) diagnostic tools can be beneficial to environmental governance^7^. Additionally, based on the availability of online sensor data collection as real-time data monitoring, with the help of citizen participation management and environmental governance, it offers a noble scientific philosophy for government decision-making in public ecological tracking and early warning^8,9^. Countries monitoring air quality have increased dramatically in recent years^10,11^. These infrastructure developments in air quality monitoring can be attributed to the government’s recently constructed or expanded monitoring networks and the crucial contributions of non-governmental groups and concerned citizens worldwide. Although progress has been achieved, several regions still lack air quality monitoring, necessitating that a substantial portion of the population access information necessary to manage pollution and make informed health decisions.

### Literature review

Deep learning (DL) and machine learning (ML) models have significantly contributed to recent developments in air quality monitoring and forecasting. These models have demonstrated extraordinary potential in predicting air pollution levels and identifying contamination sources^12,13^.

The co-training framework for air quality monitoring proposed for real-time monitoring in Beijing and Shanghai is noteworthy. By integrating spatial and temporal classifications such as artificial neural networks (ANN) and conditional random fields (CRF), this approach outperformed traditional models such as decision trees and linear interpolation. The use of real-time meteorological and traffic flow data proved beneficial in improving accuracy^14^. The key technological advancement here was the integration of various data sources for real-time monitoring. However, a significant challenge remains in the complex integration of these models, particularly when scaling to larger urban areas.

In addition, the use of wavelet-ANN models for short-term air pollution forecasting in Xi’an and Lanzhou was a key factor. The wavelet-ANN (WANN) model demonstrated superior performance in predicting air pollution indices (API), providing a higher R-value (0.8906) than traditional ANN models. This improvement was attributed to the WANN’s ability to capture non-linear patterns in pollution data. However, a challenge was the computational cost and the complexity of processing large datasets^15^.

The use of mobile air quality monitoring systems has increased, particularly through a study in Beijing, where electric vehicles equipped with real-time sensors collected PM_2.5_ data. These mobile sensors were mapped using decision tree models, which significantly outperformed fixed monitoring stations. The advantage of mobile sensors is their ability to provide high-resolution air quality data, but this approach is a challenge in fleet management and sensor calibration across a wide range^16^.

In terms of cost-effective solutions, the Deep-MAPS framework utilized mobile and fixed air quality sensors to estimate PM_2.5_ concentrations, delivering results at a resolution of 1 km × 1 km and 1 h. This model reduced hardware costs by up to 90% compared to conventional fixed sensor methods, providing a more economical way to monitor urban air quality. However, the challenges persist in ensuring secure network coverage and expanding the sensor network for broader geographic coverage^17^.

The MCST-Tree model proposed for space–time learning of air quality in Chengdu included both mobile and fixed sensor data, achieving a high accuracy (R^2^ = 0.94 for PM_2.5_) even with sparse data. This model is capable of handling space–time data gaps, but ensuring high model accuracy with limited sensor data is an ongoing challenge^18^. In Chengdu, the Multi-AP learning system was introduced for high-resolution pollutant mapping. This method produced detailed hourly pollution maps, resulting in a decrease in computational efficiency and accuracy. However, the challenge remains to maintain the complexity of real-time predictions, particularly for large-scale urban areas^19^.

For long-term forecasting, an ANN-based model for PM_2.5_ concentrations in Liaocheng demonstrated a high accuracy (R = 0.9570), resulting in Bayesian regularization. The challenge of mitigating overfitting and guaranteeing consistent performance across a variety of conditions persists, although this approach was successful in long-term predictions^20^. In forecasting volatility, a hybrid XGBoost-GARCH-MLP model was employed for PM_2.5_ volatility prediction, providing better long-term prediction accuracy. The hybrid model’s strength was in incorporating volatility into the forecasting process, yet the complexity and high computational requirements of such models are limited to their practical application^21^. In addition, AI-based models such as ANN, CNN, and LSTM have been employed for climate and air quality forecasting in cities such as Jinan and Hohhot, where CNN-LSTM models showed superior performance. These multimodal forecasting techniques provide a great opportunity to improve air quality predictions, though challenges persist in model generalization and integrating across diverse regions^22^. ST-Exposure, a promising model, utilizes fixed and mobile sensors to predict PM_2.5_ exposure on a pixel-wise basis. This model achieved an SMAPE below 15%, indicating its potential in high-resolution exposure predictions. However, the challenges of sparse sensor deployment and data integration remain obstacles to achieving optimal accuracy^23^.

While significant efforts have been made in utilizing ML and DL techniques for air quality forecasting, data quality, computational complexity, model integration, and coverage persist. Future advances in sensor technology, data fusion, and model optimization will be crucial in advancing these methods for broader geographical applications and improving their practical application.

### Research objectives and novelty

While deep learning models such as ANN, CNN, and LSTM have shown strong performance in predicting air pollution, each comes with its own set of challenges. ANN models are susceptible to overfitting and often struggle to generalize across different geographical areas. CNNs are good at identifying spatial features, but they typically need large datasets and may fail to capture time-based patterns. LSTMs handle temporal data well, but they require significant computational resources and can perform poorly when data is noisy or incomplete. Additionally, many deep learning approaches have trouble integrating diverse data types—like weather, traffic, and sensor inputs—and scaling efficiently in complex urban settings. These limitations point to the importance of enhanced data preprocessing, hybrid model approaches, and transfer learning techniques. Therefore, the research is aimed at the forecasting problems of six fundamental pollutants—Carbon Monoxide (CO), Nitrogen Dioxide (NO_2_), Ozone (O_3_), Sulphur Dioxide (SO_2_), Fine Particulate Matter (PM_2.5_), and Coarse Particulate Matter (PM_10_) using state-of-the-art machine learning approaches, specifically Deep Neural Networks (DNNs) and Convolutional Neural Networks (CNNs). These pollutants are particularly important due to their adverse effects on the environment and the environment.

The research uses data from the Beijing Municipal Environmental Monitoring Centre (BMEMC) from March 2013 to February 2017, which includes meteorological data and pollutant levels from 10 nationally controlled monitoring sites. This study intends to provide hourly predictions that enable a more accurate assessment of the health-related impacts of air pollution, unlike the traditional models that often provide poor temporal accuracy.

This study is a unique approach to air pollution prediction using DNNs and CNNs based on multivariate time series analysis. Until now, only a few ML studies have attempted to apply such high temporal resolution to pollutant concentration prediction using feature-encoded DNN and CNN frameworks with both encoded and unencoded features. The contribution captures intricate spatiotemporal structures that especially tend to be masked in strongly polluted urban settings where both time and pollution levels fluctuate dramatically. The study utilizes sophisticated pre-processing of data, including interpolation of missing values, as well as thorough exploratory analysis using box plots to ensure the accuracy and integrity of the data. This strategy enhances the accuracy of air quality predictions and the model’s scalability for real-time forecasting; likewise, urban planners and public health regulators can monitor hourly pollutant concentrations in their areas to implement effective pollution management methods. This work aims to provide reliable emission prediction models that could guide decisions regarding human health protection and environmental sustainability.

## Study area and data

In January 2013, Beijing established 36 air-quality monitoring sites, 35 of which are Beijing Municipal Environmental Monitoring Center (BMEMC) sites, and one at the US Embassy in Beijing^24^. The current dataset comprises hourly air pollutant data from 10 national air quality monitoring stations, namely Aotizhongxin, Changping, Dongsi, Guanyuan, Huairou, Nongzhanguan, Shunyi, Tiantan, Wanliu, and Wanshouxigong. These ten stations were chosen due to the availability of free access to their data. The meteorological data in each air-quality site are compared to the nearest weather station from the China Meteorological Administration (CMA). The data was recorded in hours from 01/03/2013 to 28/02/2017. The datasets included four time attributes such as (year of the data, month of the data, day of the data, and hour of the data), six principal air pollutants such as (PM_2.5_ concentration (ug/m^3^), PM_10_ concentration (ug/m^3^), SO_2_ concentration (ug/m^3^), NO_2_ concentration (ug/m^3^), CO concentration (ug/m^3^) and O_3_ concentration (ug/m^3^)), and six relevant meteorological variables such as (temperature (°C), pressure (hPa), dew point temperature (°C), rainfall (mm), wind direction and wind speed (m/s)).

## Artificial intelligence models

### Deep neural networks (DNN)

Artificial neural networks (ANNs) are an effective machine learning technique based on the human brain’s structure. In self-learning, ANNs can identify patterns and hidden correlations in datasets^25^. Furthermore, a particular type of ANN called a deep neural network (DNN) has numerous layers of connected nodes, which enables it to represent more complex data relationships and perform better than traditional ANNs^26^.

**Fig. 1:**
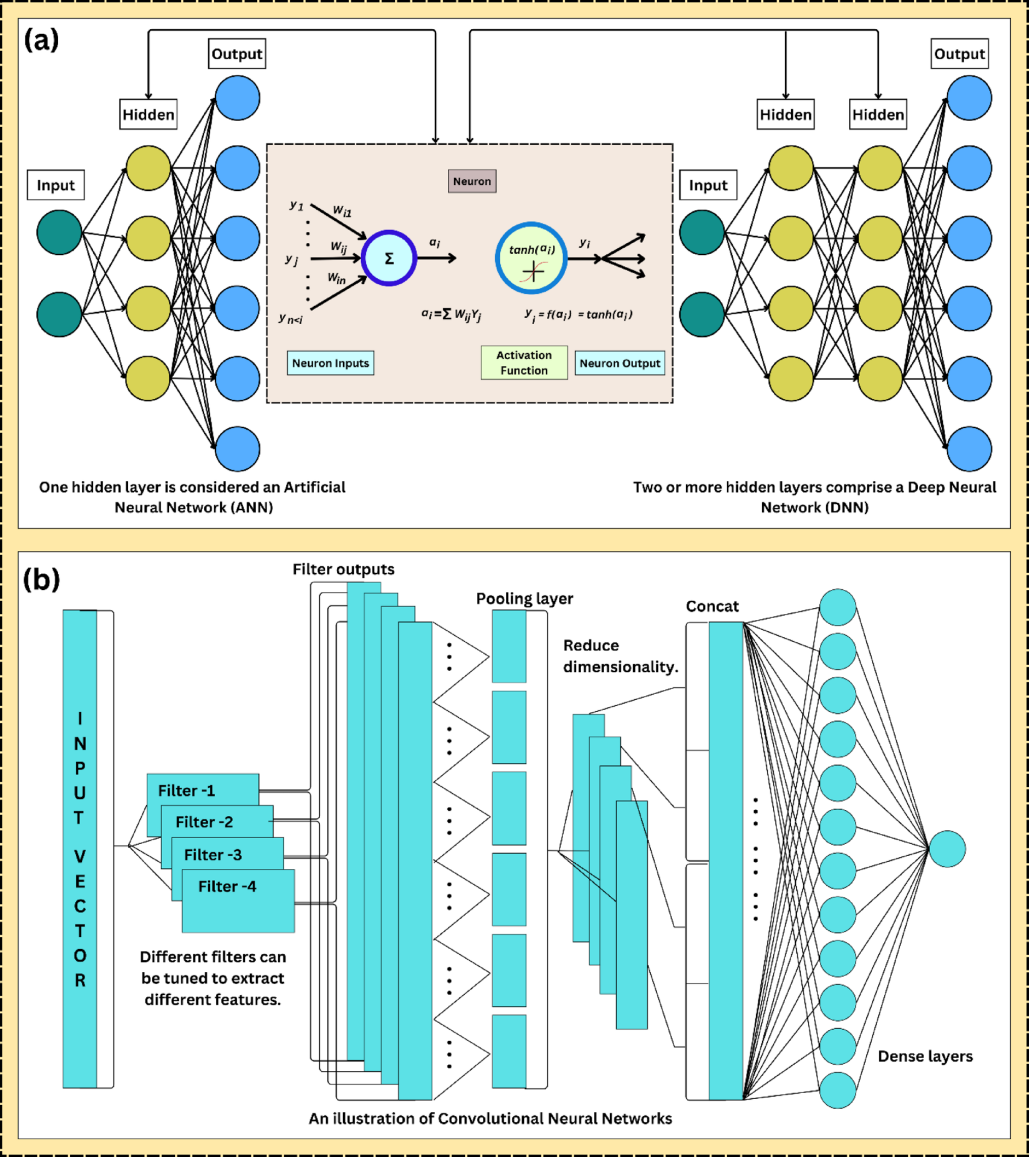
Schematic diagrams of deep learning models; (**a**) DNN, (**b**) CNN.

The layers in the DNN model typically have an input layer, three or more hidden layers, and an output layer. The input layer receives the data, which is then altered by the hidden layers, and the output layer generates a forecast. Figure 1a displays the DNN model’s architecture employed in this investigation. A single neuron receives inputs, multiplies each input by the corresponding weight ($W$), adds a bias ($b$), and then passes the sum through an activation function ($f(x)$) to produce an output. The weights and biases are calculated to determine the impact of the inputs. At the same time, the activation function provides nonlinearity and enables the model to learn complex patterns^27^.

A backpropagation approach is employed to alter the weights between the nodes to train a DNN model. This strategy eliminates the disparity between the forecasted and actual output by altering the weights until the network can reliably predict new data. A DNN model can identify complex relationships in data and generate precise predictions when given new data, if it receives sufficient training^28^.

### Convolutional neural network (CNN)

The CNN network’s convolutional and pooling layers are the essential elements of feature extraction^29^. Time series data are typically the primary application for 1D-CNNs due to their strong feature extraction capabilities^30^. Alternating convolutional and pooling layers in the 1D-CNN enable the extraction of non-linear features from raw data, and the fully connected layer completes adaptive feature learning^31^.

The basic architecture of the CNN is outlined in Fig. 1b, which comprises an input layer, several convolutional layers, several pooling layers, a fully connected layer, and an output layer. The convolutional and pooling layers are connected in an alternating fashion. In this regard, the CNN feature extraction module comprises the input, convolutional, and pooling layers. The output module includes the connected and output layers^32^. The complete calculation formula is outlined in Eq. (1).

1 $$y_{j} = f\left( {\mathop \sum \limits_{{i \in M_{j} }} x_{i}^{l - 1} \otimes w^{l}_{i,j} + b^{l}_{j} } \right)$$

where, $f$ is the activation function, $\otimes$ is the convolutional operator, $w$ is the weight matrix, and $b$ is the bias deviation.

### Model development and configuration

Ten nationally controlled air-quality monitoring sites—Aotizhongxin, Changping, Dongsi, Guanyuan, Huairou, Nongzhanguan, Shunyi, Tiantan, Wanliu, and Wanshouxigong—provided hourly air quality data for this study. The dataset spans from March 1, 2013, to February 28, 2017, and includes four temporal attributes (year, month, day, and hour), six major air pollutants (PM_2.5_, PM_10_, SO_2_, NO_2_, CO, and O_3_ in µg/m^3^), and six meteorological variables (dew point temperature (°C), air temperature (°C), pressure (hPa), wind direction, wind speed (m/s), and precipitation (mm)).

In preprocessing, each station’s dataset was analyzed to identify and impute missing values using linear interpolation (Table 1). The final cleaned dataset contained 35,064 instances per station. These were split into training, validation, and testing sets with a time-series window size of 10, resulting in: Training: (25,000, 10, number of features), (25,000), Validation: (5000, 10, number of features), (5000) and Testing: (5054, 10, number of features), (5054).

**Table 1:** Distribution of missing values (NaN) across columns in multiple stations.

	PM_2.5_	PM_10_	SO_2_	NO_2_	CO	O_3_	Temp	Pres	Dewp	Rain	WD	WS
Aotizhongxin	925	718	935	1023	1776	1719	20	20	20	20	81	14
Changping	774	582	628	667	1521	604	53	50	53	51	140	43
Dongsi	750	553	663	1601	3197	664	20	20	20	20	78	14
Guanyuan	616	429	474	659	1753	1173	20	20	20	20	81	14
Huairou	953	777	980	1639	1422	1151	51	53	53	55	302	49
Nongzhanguan	628	440	446	692	1206	506	20	20	20	20	78	14
Shunyi	913	548	1296	1365	2178	1489	51	51	54	51	483	44
Tiantan	677	597	1118	744	1126	843	20	20	20	20	78	14
Wanliu	382	284	575	1070	1812	2107	20	20	20	20	123	14
Wanshouxigong	696	484	669	754	1297	1078	19	19	19	19	79	13

**Fig. 2:**
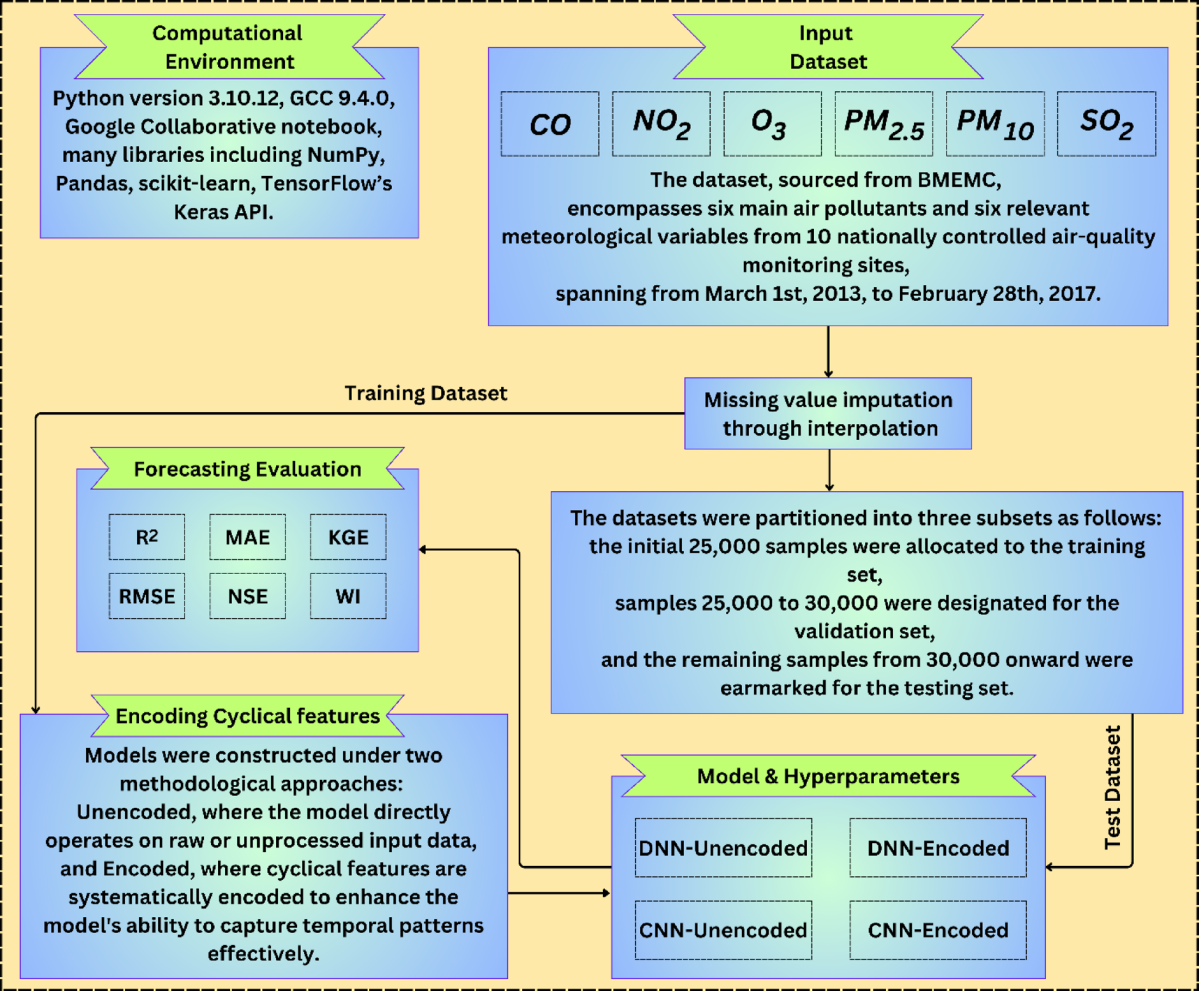
Model development and configuration for forecasting hourly air pollutants in 10 Beijing stations.

To improve prediction accuracy (Fig. 2), the proposed method incorporates both **temporal** and **spatial** features. Temporal features (e.g., hour, day, month) are inherently cyclical. To model this periodicity, each cyclical feature was encoded using sine and cosine transformations, allowing the model to capture repeating patterns. Specifically Eqs. (2–4):

2 $$hou{r}_{sin}=sin\left(\frac{2\pi .hour}{24}\right), hou{r}_{cos}=cos\left(\frac{2\pi .hour}{24}\right)$$

3 $${day}_{sin}=sin\left(\frac{2\pi .day}{31}\right), {day}_{cos}=cos\left(\frac{2\pi .day}{31}\right)$$

4 $${month}_{sin}=sin\left(\frac{2\pi .month}{24}\right), {month}_{cos}=cos\left(\frac{2\pi .month}{24}\right)$$

These transformations help preserve the cyclical continuity (e.g., hour 23 to hour 0) and support the model in learning seasonal or diurnal effects, especially during transitions such as dawn/dusk or seasonal changes.

Spatial features are derived from the geographical and industrial characteristics unique to each monitoring site. These include proximity to traffic, industrial zones, and residential areas, which introduce local dependencies into pollution patterns. Rather than generalizing across all locations, the model trains separately for each station to account for such location-specific dynamics.

The rationale for selecting **DNN** and **CNN** lies in their respective strengths:

i. DNNs are effective for learning non-linear feature interactions, especially when the dataset includes mixed data types (e.g., meteorological and pollutant data).
ii. CNNs are chosen for their ability to capture local patterns across the time dimension, as convolutional filters can detect trends and abrupt changes in short sequences—an important feature in hourly air pollution data.

Both models were implemented for each station independently to capture station-specific pollution dynamics. Furthermore, in both models, we set window_size = 10, which refers to the number of consecutive time steps (or rows) used to construct a single input sample for the time series forecasting model. The encoded approach outperformed the unencoded baseline in capturing temporal fluctuations and spatial heterogeneity. This approach led to improved air quality forecasting accuracy and provided insights into region-specific pollution trends. As per Table 2, to ensure fair and optimal performance of both DNN and CNN architectures across unencoded and encoded feature sets, a systematic hyperparameter tuning process was conducted using grid search and fivefold cross-validation on the training data, aiming to minimize validation RMSE. The model architectures were designed to balance complexity and generalization, with DNNs using three dense layers (32 → 16 → 8 units) for progressive feature abstraction and CNNs employing a kernel size of 2 with 32 filters for efficient local pattern extraction. The Adam optimizer with a learning rate of 0.005—selected from the range {0.001, 0.003, 0.005, 0.01}—offered stable convergence and the lowest validation error. MSE was used as the loss function, while RMSE served as the evaluation metric for its interpretability. Models were trained for 100 epochs, with early stopping applied to prevent overfitting.

**Table 2:** Components and descriptions for DNN and CNN models with Unencoded and Encoded features.

Model type	Component	Description
DNN (Unencoded)	Input layer	Shape: (10, 10)
	Flatten layer	Flatten the input to a 1D array
	Hidden layers	Dense layer 1: Units = 32, Activation = ReLU
		Dense layer 2: Units = 16, Activation = ReLU
		Dense layer 3: Units = 8, Activation = ReLU
	Output layer	Units: 1, Activation: Linear
	Training configuration	Loss Function: (MSE)
		Optimizer: Adam
		Learning Rate: 0.005
		Metrics: (RMSE)
		Epochs: 100
CNN (Unencoded)	Input layer	Input Shape (10, 10)
	Convolution layer	Filters: 32
		Kernel Size: 2
		Activation: ReLU
	Flattening layer	Flattens the input data
	Hidden layers	Dense Layer 1: Units = 16, Activation = ReLU
		Dense Layer 2: Units = 8, Activation = ReLU
	Output layer	Units = 1, Activation = Linear
	Training configuration	Loss Function: (MSE)
		Optimizer: Adam
		Learning Rate: 0.005
		Metrics: (RMSE)
DNN (Encoded)	Input layer	Shape: (10, 7)
	Flatten layer	Flatten the input to a 1D array
	Hidden layers	Dense layer 1: Units = 32, Activation = ReLU
		Dense layer 2: Units = 16, Activation = ReLU
		Dense layer 3: Units = 8, Activation = ReLU
	Output layer	Units: 1, Activation: Linear
	Training configuration	Loss Function: (MSE)
		Optimizer: Adam
		Learning Rate: 0.005
		Metrics: (RMSE)
		Epochs: 100
CNN (Encoded)	Input layer	Input Shape (10, 7)
	Convolution layer	Filters: 32
		Kernel Size: 2
		Activation: ReLU
	Flattening layer	Flattens the input data
	Hidden layers	Dense Layer 1: Units = 16, Activation = ReLU
		Dense Layer 2: Units = 8, Activation = ReLU
	Output layer	Units = 1, Activation = Linear
	Training configuration	Loss Function: (MSE)
		Optimizer: Adam
		Learning Rate: 0.005
		Metrics: (RMSE)

### Forecasting metrics

In this paper, the following metrics were applied to measure the efficiencies of unencoded and encoded deep learning models:

i. Mean Absolute Error (MAE)
  5 $$MAE=\frac{1}{N}\times \sum_{i=1}^{N}\left|{P}_{i}-{O}_{i}\right|$$
ii. Mean Squared Error (MSE)
  6 $$MSE=\frac{1}{N}\times \sum_{i=1}^{N}{\left({P}_{i}-{O}_{i}\right)}^{2}$$
iii. Root Mean Square Error (RMSE)
  7 $$RMSE=\sqrt{MSE}=\sqrt{\frac{1}{N}\times \sum_{i=1}^{N}{\left({P}_{i}-{O}_{i}\right)}^{2}}$$
iv. Coefficient of Determination (R^2^)
  8 $${R}^{2}= 1-\frac{\sum {\left({P}_{i}-{O}_{i}\right)}^{2}}{\sum {\left({P}_{i}-{O}_{i}\right)}^{2}}$$
v. Willmott Index (WI)
  9 $$WI= 1-\frac{{\sum }_{i=1}^{N}{({O}_{i}-{P}_{i})}^{2}}{{\sum }_{i=1}^{N}{(\left|{P}_{i}-\overline{O }\right|+\left|{O}_{i}-\overline{O }\right|)}^{2}}$$
vi. Kling-Gupta Efficiency (KGE)
  10 $$KGE= 1-\sqrt{{\left(PCC-1\right)}^{2}+{(\frac{std}{rd}-1)}^{2}+(\frac{\overline{O} }{\overline{P} }-1)}$$

In the above equations, ${O}_{i}$ is the observed (actual) value of air pollutants. ${P}_{i}$ is the forecasted value of air pollutants. $\overline{O }$ and $\overline{P }$ are the average values of the observed and forecasted values of air pollutants, respectively. $PCC$, $std,$ and $rd$ are the Pearson correlation coefficient, the standard deviation of forecasted values, and the standard deviation of observation values, respectively.

## Results and discussion

### Aotizhongxin station

**Fig. 3:**
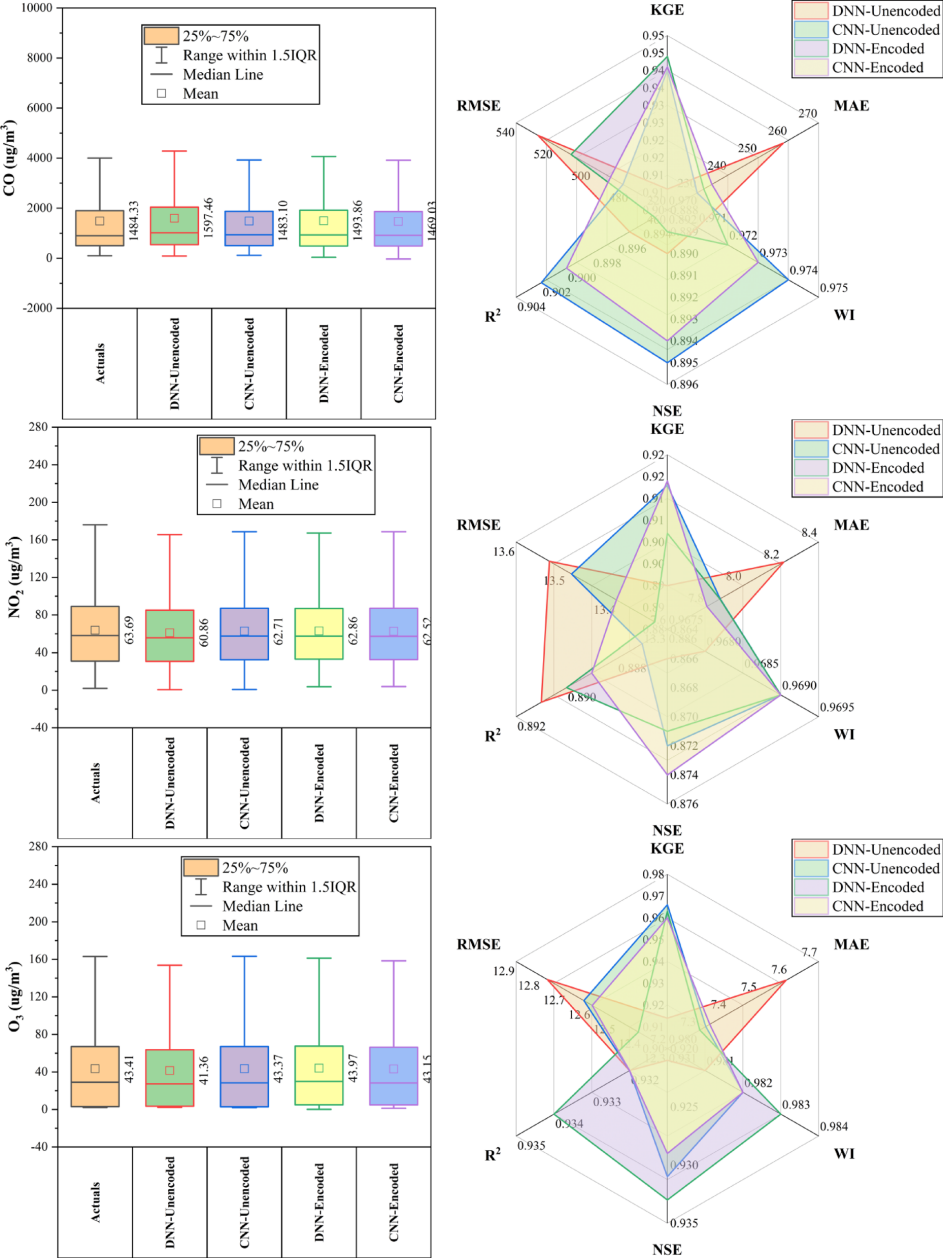

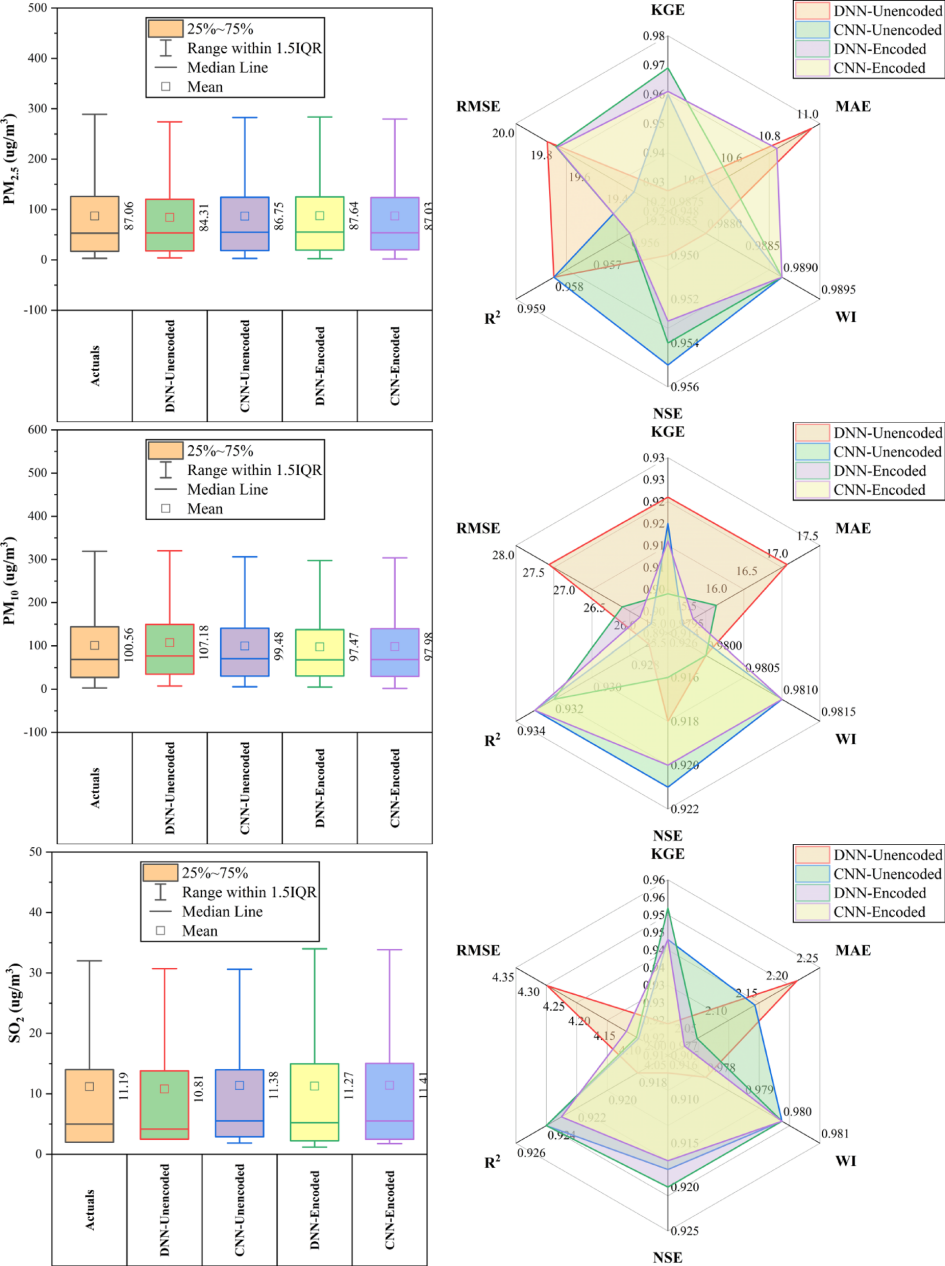
Forecasting results of six air pollutants using Unencoded and Encoded deep learning models at Aotizhongxin Station.

At Aotizhongxin station (Fig. 3), DNN and CNN models showed distinct strengths across pollutants. CNN-Unencoded performed best for CO (RMSE: 483.5 µg/m^3^) and PM_10_ (KGE: 0.921), while DNN-Encoded led in NO_2_ (KGE: 0.914), O_3_ (RMSE: 12.4 µg/m^3^), and SO₂ (KGE: 0.952). The dew point was the dominant predictor in ~ 83% of top-performing models. Rainfall and hourly features contributed minimally (< 10%). PM_2.5_ forecasts exhibited the highest variability. Overall, performance was driven by broad temporal and environmental patterns, with each model excelling in specific pollutant contexts.

### Changping station

**Fig. 4:**
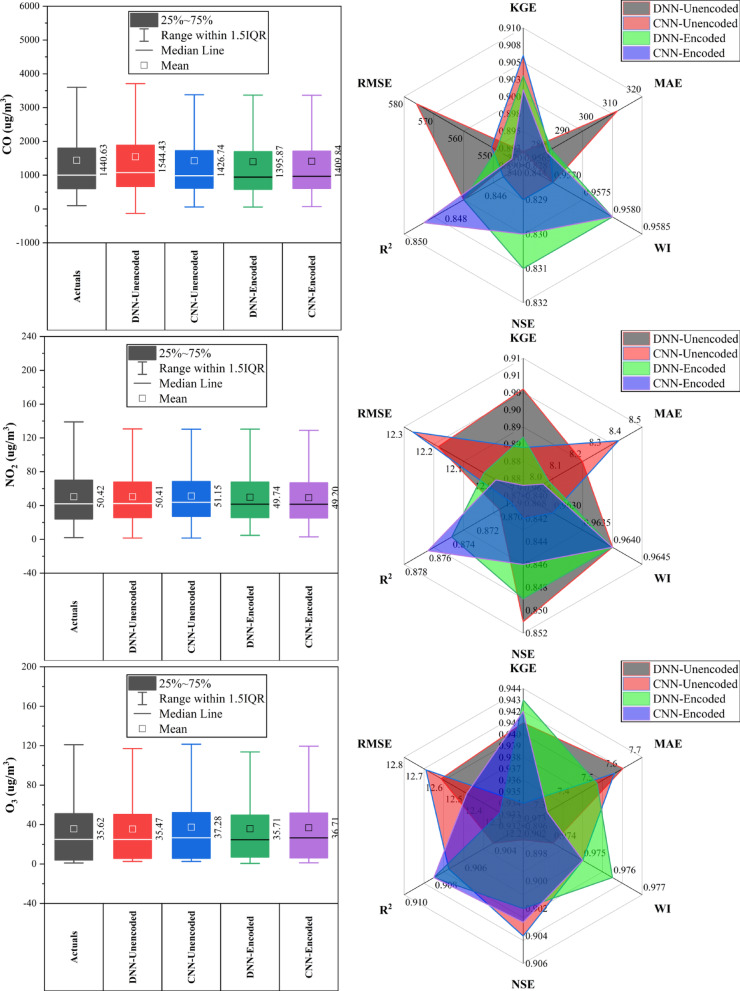

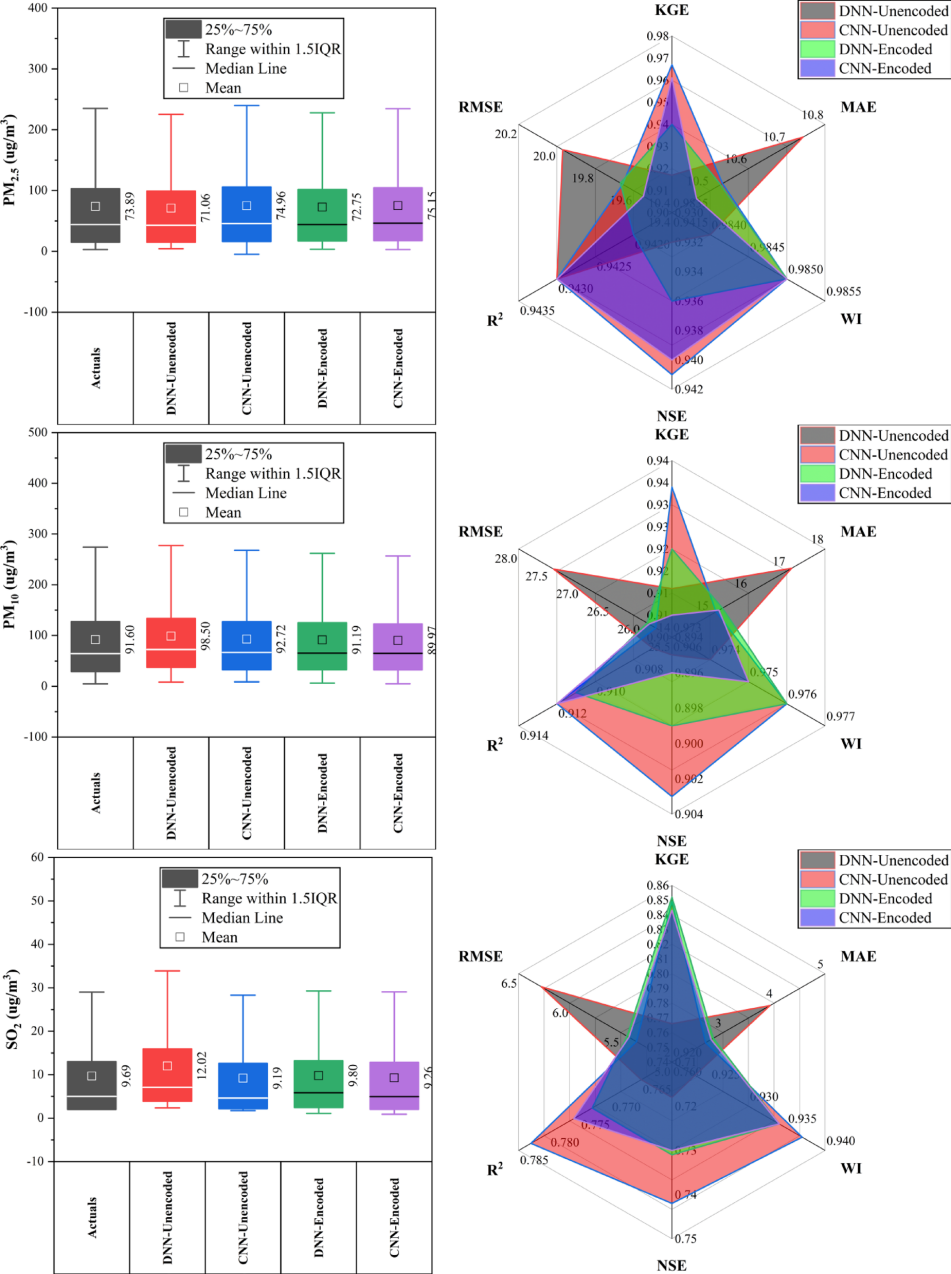
Forecasting results of six air pollutants using Unencoded and Encoded deep learning models at Changping Station.

At Changping Station (Fig. 4), CNN-Encoded models showed consistently strong performance across pollutants. They achieved the lowest MAE and RMSE in 67% of cases and ranked highest in R^2^ or KGE for ~ 50%. For CO, CNN-Encoded had R^2^ = 0.849 and MAE = 279.9 µg/m^3^, outperforming CNN-Unencoded despite a slightly lower KGE. In NO_2_, CNN-Encoded reduced MAE by ~ 21% compared to DNN-Unencoded. O_3_ forecasts showed close performance: DNN-Encoded had the highest KGE (0.943), while CNN-Encoded achieved the lowest MAE (7.3 µg/m^3^). For PM_2.5_ and PM_10_, CNN-Unencoded slightly outperformed in KGE and R^2^, but CNN-Encoded had lower error metrics. SO_2_ results were mixed, with CNN-Unencoded leading in R^2^ (0.983), while DNN-Encoded topped in KGE (0.852). Dew point, month_cosine, and month were key predictors in over 80% of models, while rainfall and hourly features had < 10% impact.

### Dongsi station

**Fig. 5:**
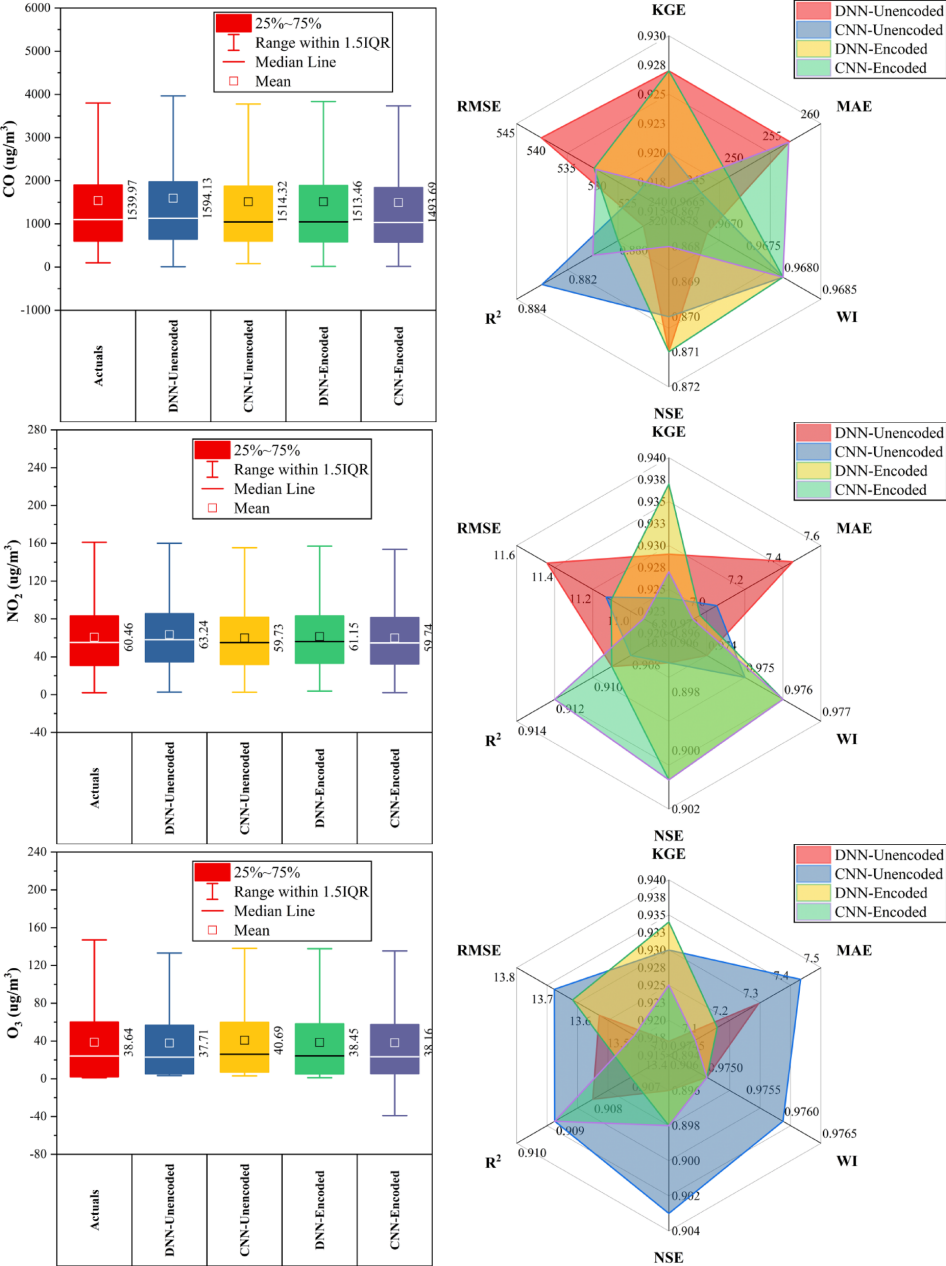

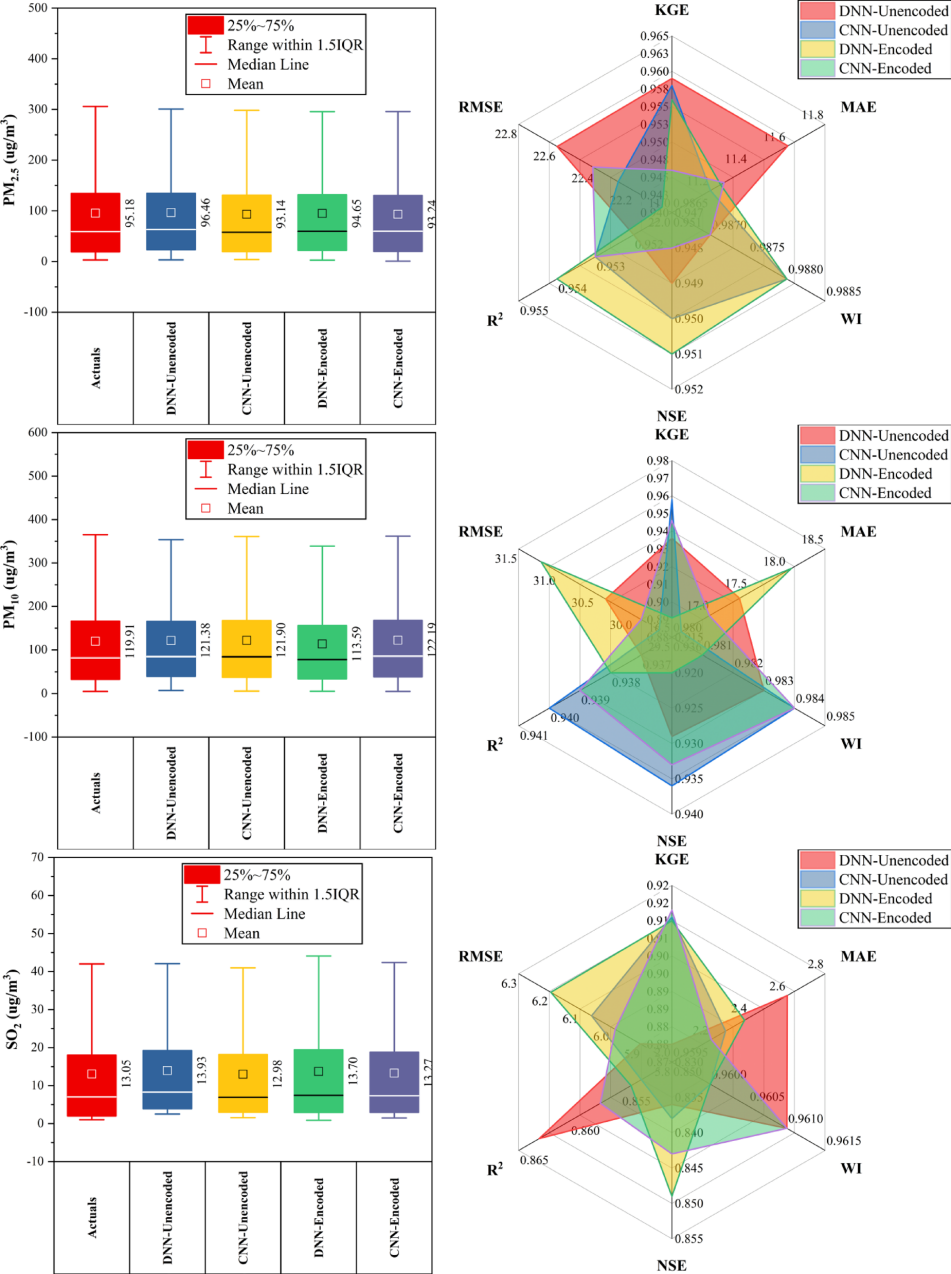
Forecasting results of six air pollutants using Unencoded and Encoded deep learning models at Dongsi Station.

At Dongsi Station (Fig. 5), CNN models—particularly CNN-Encoded—demonstrated superior forecasting accuracy for most pollutants. CNN-Encoded achieved the lowest MAE and RMSE in 60% of pollutants, excelling in CO (MAE: 244.2 µg/m^3^), NO_2_ (MAE: 6.93 µg/m^3^; R^2^: 0.912), and SO_2_ (MAE: 2.21 µg/m^3^). CNN-Unencoded led in KGE and WI for PM_2.5_ (KGE: 0.959) and PM_10_ (KGE: 0.934), while DNN-Encoded had the lowest RMSE for PM_2.5_ (22.05 µg/m^3^) and the highest KGE for O_3_ (0.934). Dew point, month_cosine, and temperature were key predictors in > 80% of cases, while rainfall had < 10% influence. Overall, CNN models outperformed others in MAE/RMSE in most cases, confirming their robustness in pollutant forecasting.

### Guanyuan station

**Fig. 6:**
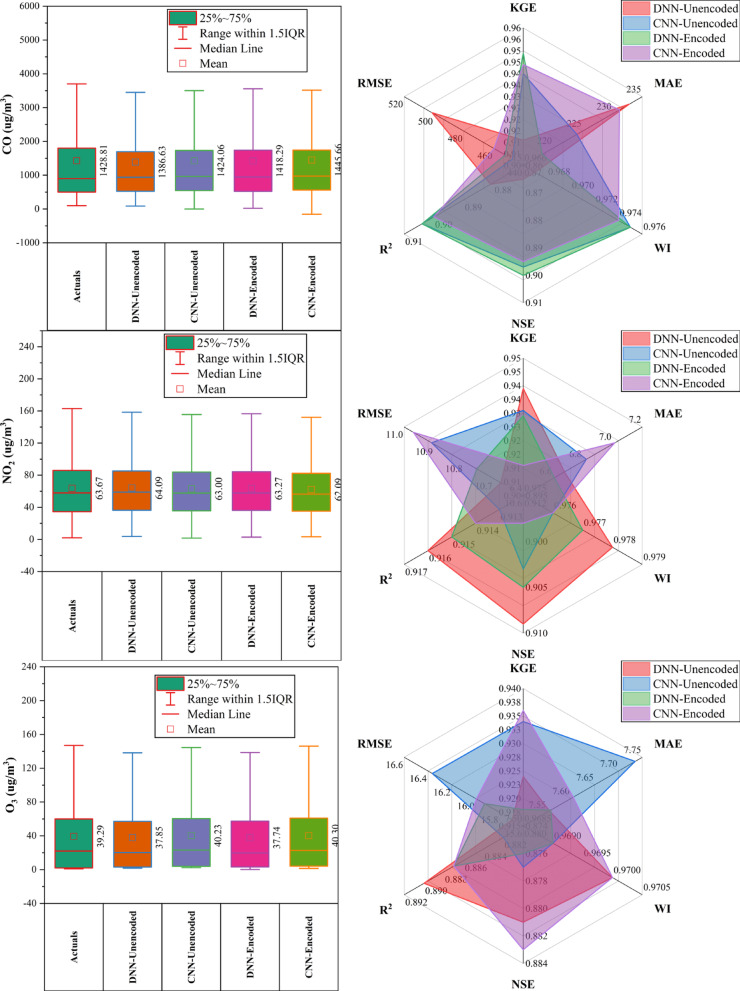

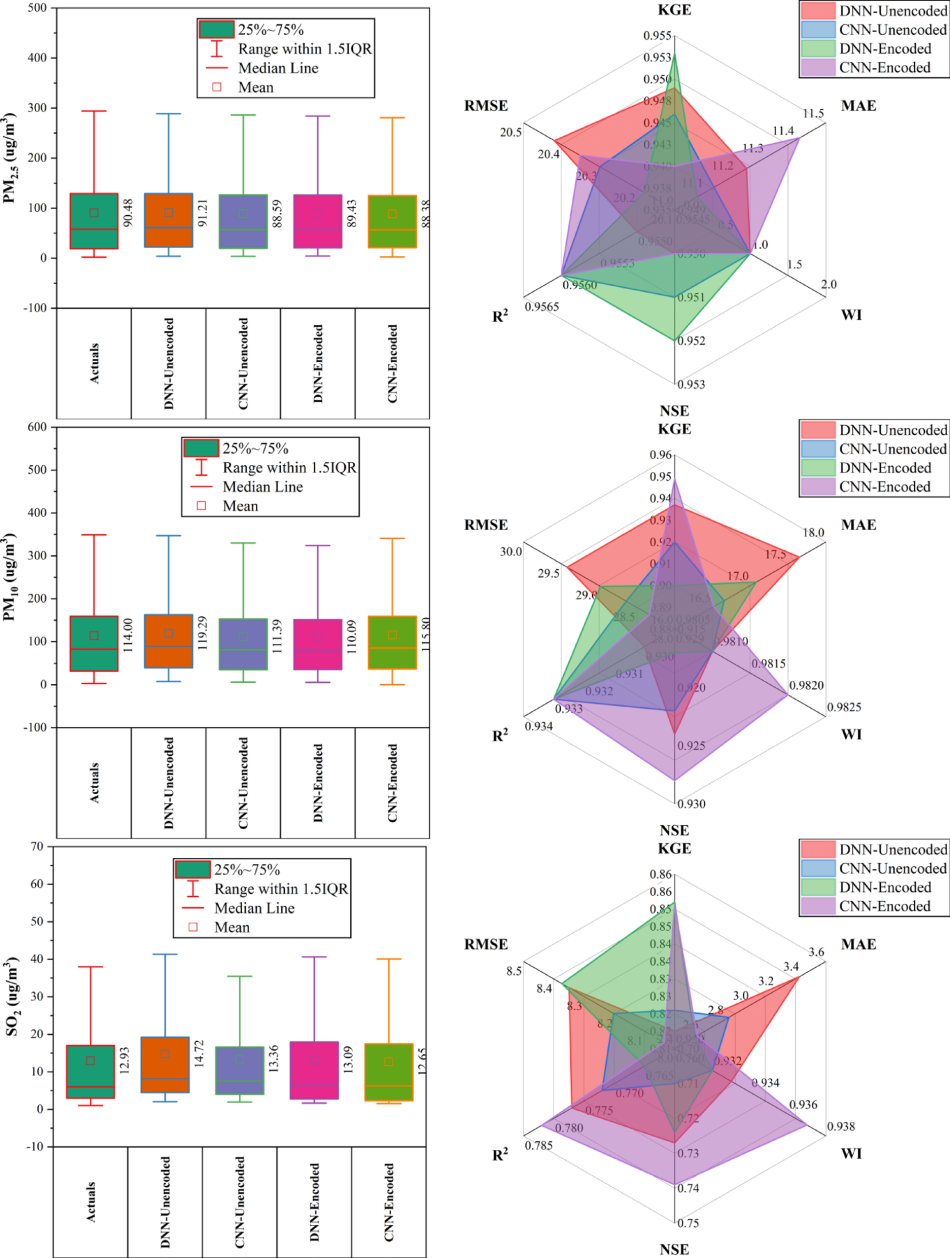
Forecasting results of six air pollutants using Unencoded and Encoded deep learning models at Guanyuan Station.

At Guanyuan Station (Fig. 6), DNN-Encoded models outperformed in 60% of pollutants, achieving top KGE and NSE for CO (KGE: 0.949; NSE: 0.9), PM_2.5_, and SO_2_, along with the lowest MAE/RMSE for CO and PM_2.5_. CNN-Encoded led in O_3_ (KGE: 0.936; WI: 0.97) and PM_10_ (KGE: 0.949; WI: 0.982). DNN-Unencoded had the highest R^2^ for NO_2_ (0.916) and lowest MAE/RMSE for O_3_, despite lower KGE/NSE. Key predictors across models included dew point, month_cosine, and temperature (relevant in > 80% of top-performing models), while rainfall and hour_sine had < 10% impact. Overall, encoded models performed better on KGE/NSE/WI, while unencoded models excelled in MAE/RMSE for select pollutants, emphasizing pollutant-specific model suitability.

### Huairou station

**Fig. 7:**
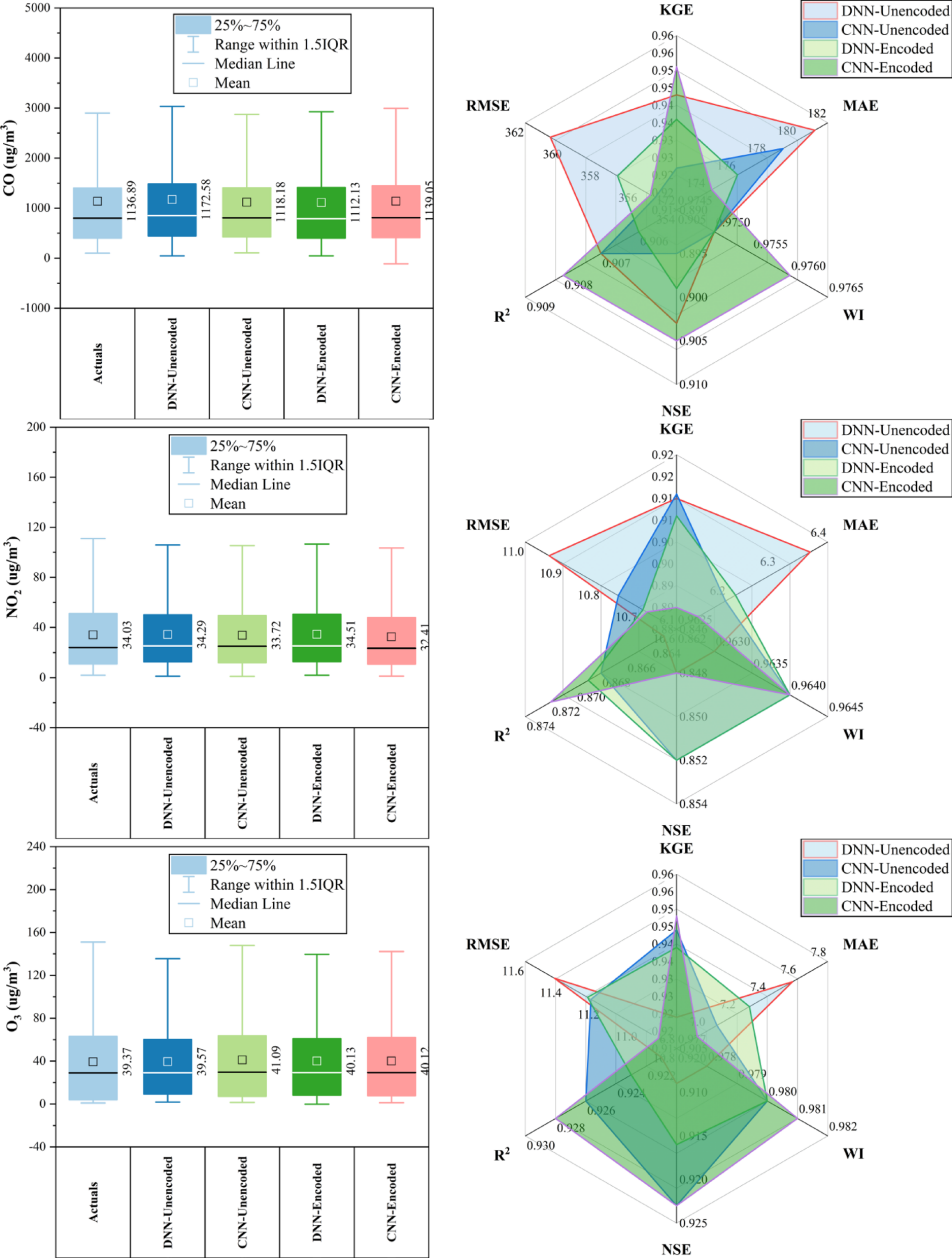

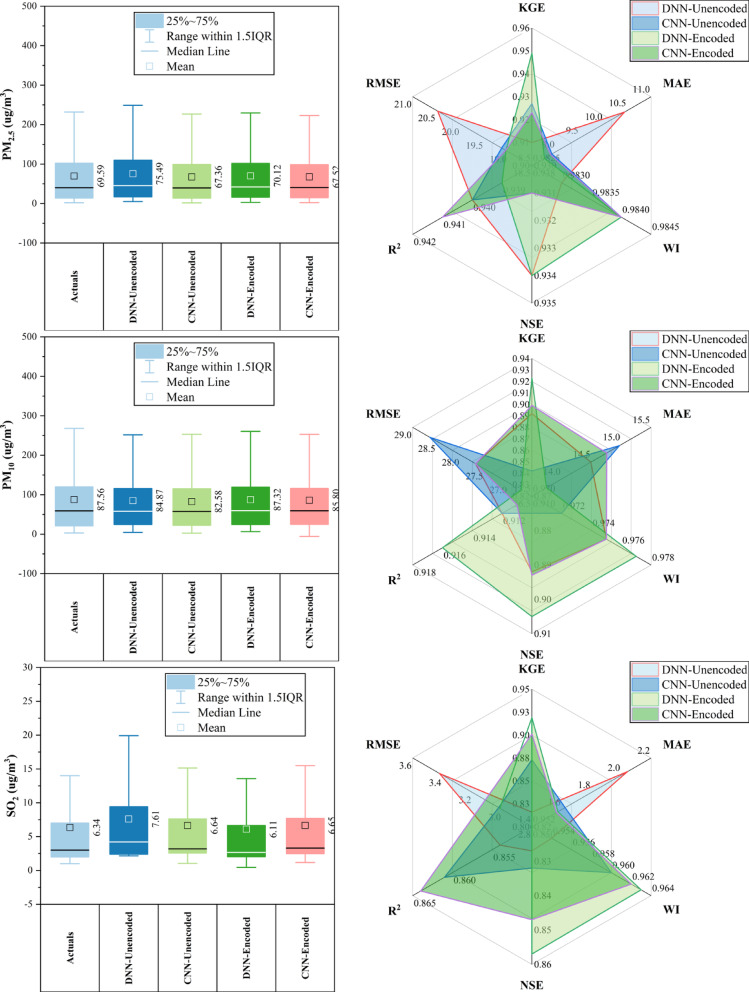
Forecasting results of six air pollutants using Unencoded and Encoded deep learning models at Huairou Station.

At Huairou Station (Fig. 7), CNN-Encoded models outperformed others in ~ 70% of pollutant forecasts, achieving top KGE (0.951) and WI (0.976) for CO, and lowest errors for CO, NO_2_, and O_3_. DNN-Encoded excelled for PM_2.5_ (KGE: 0.949), PM_10_, and SO_2_, showing better KGE and NSE in ~ 30% of cases. Encoding improved performance across all pollutants, particularly for CO, NO_2_, and O_3_. Key predictors—dew point, month, and temperature—were influential in over 80% of top-performing models, while rainfall and hourly features had minimal impact (< 10%). Overall, encoded models consistently delivered superior accuracy by effectively capturing temporal and environmental patterns.

### Nongzhanguan station

**Fig. 8:**
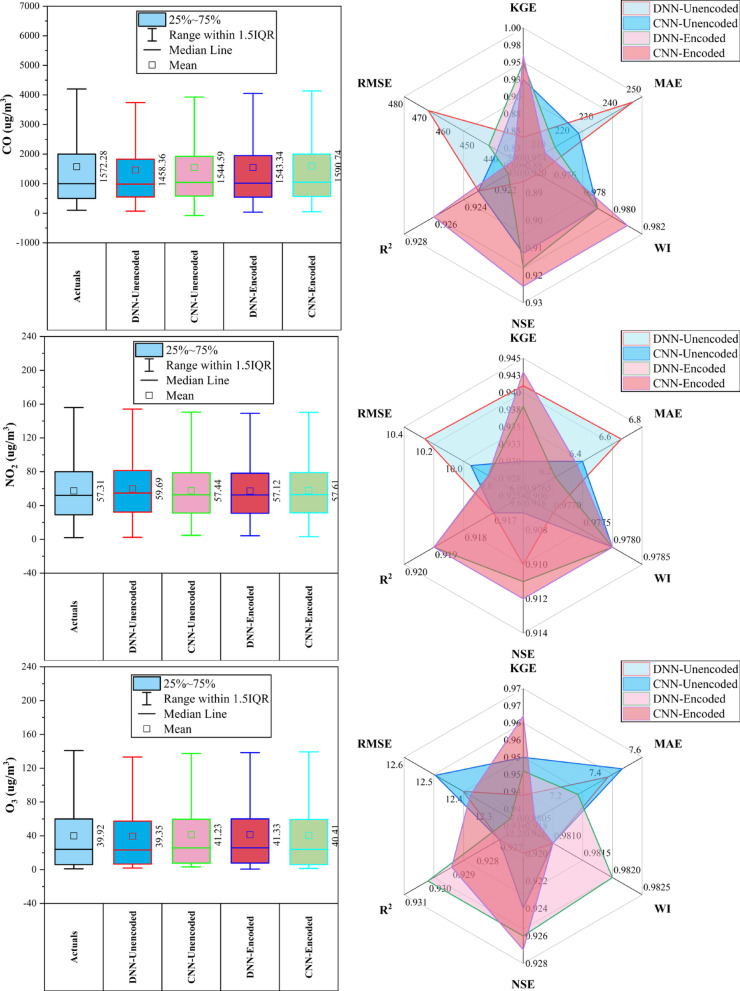

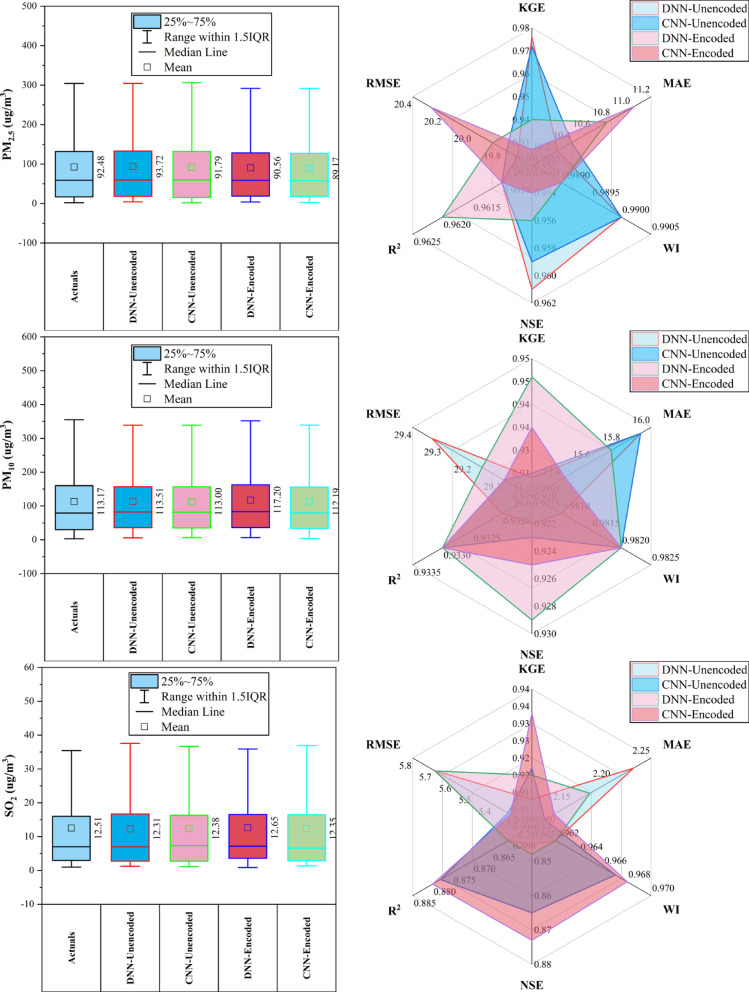
Forecasting results of six air pollutants using Unencoded and Encoded deep learning models at Nongzhanguan Station.

At Nongzhanguan Station (Fig. 8), CNN-Encoded models outperformed others in ~ 70% of pollutants, achieving top KGE for CO (0.960), O_3_ (0.962), and NO_2_ (0.943), with the lowest MAE/RMSE (e.g., CO MAE: 209.2 μg/m^3^; O_3_ MAE: 7.1 μg/m^3^). DNN-Unencoded excelled in PM_2.5_ with the highest KGE (0.976) and lowest RMSE (19.7 μg/m^3^). Forecasts for CO, NO_2_, and O_3_ had strong accuracy (KGE > 0.92; R^2^ > 0.91), while SO_2_ had the lowest performance (max KGE: 0.933; R^2^: 0.88). Dew point, temperature, and pressure were key predictors in > 80% of cases; rainfall had a < 10% impact. Overall, CNN-Encoded models proved most effective for pollutant forecasting, driven by strong meteorological and temporal features.

### Shunyi station

**Fig. 9:**
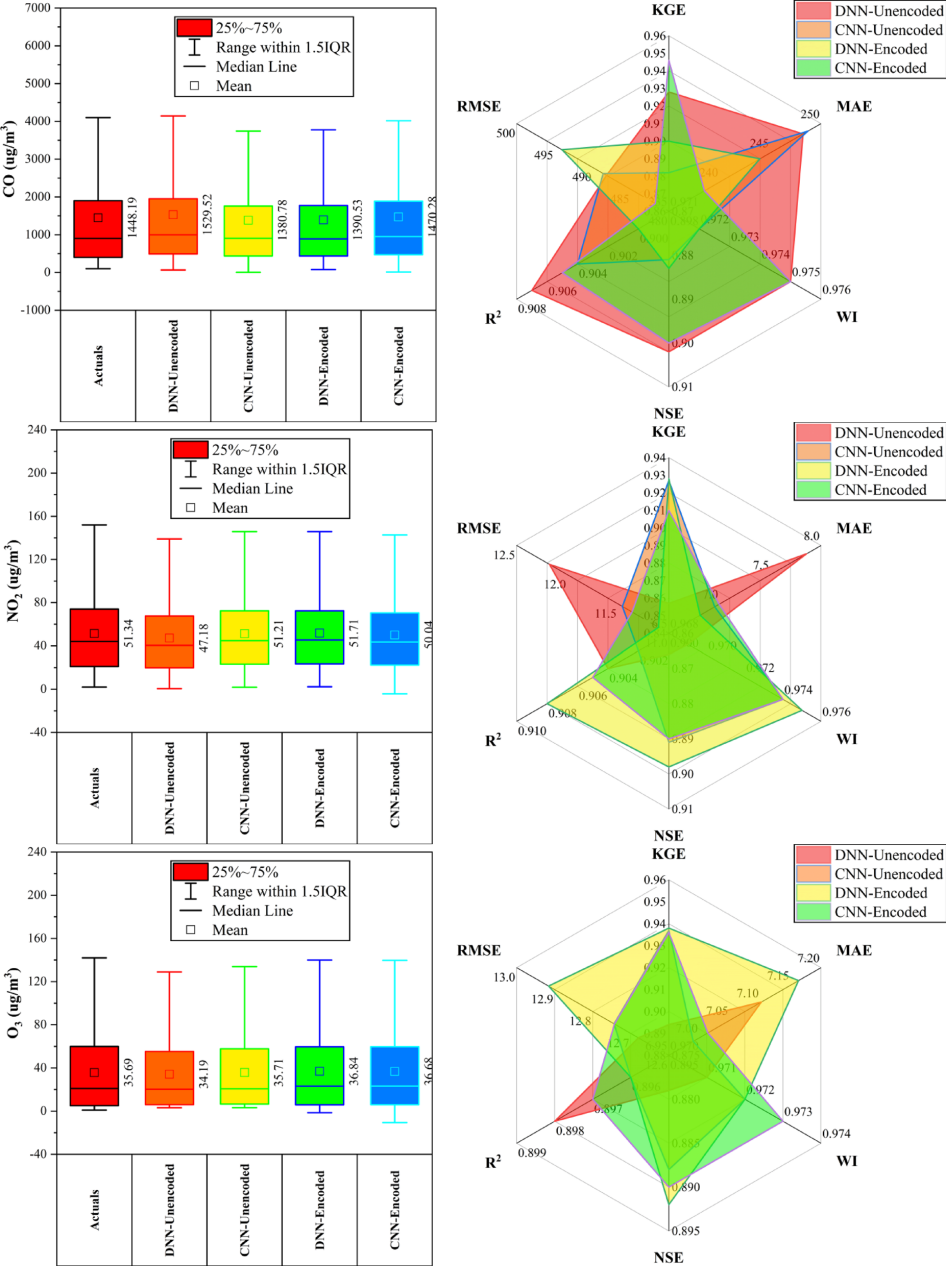

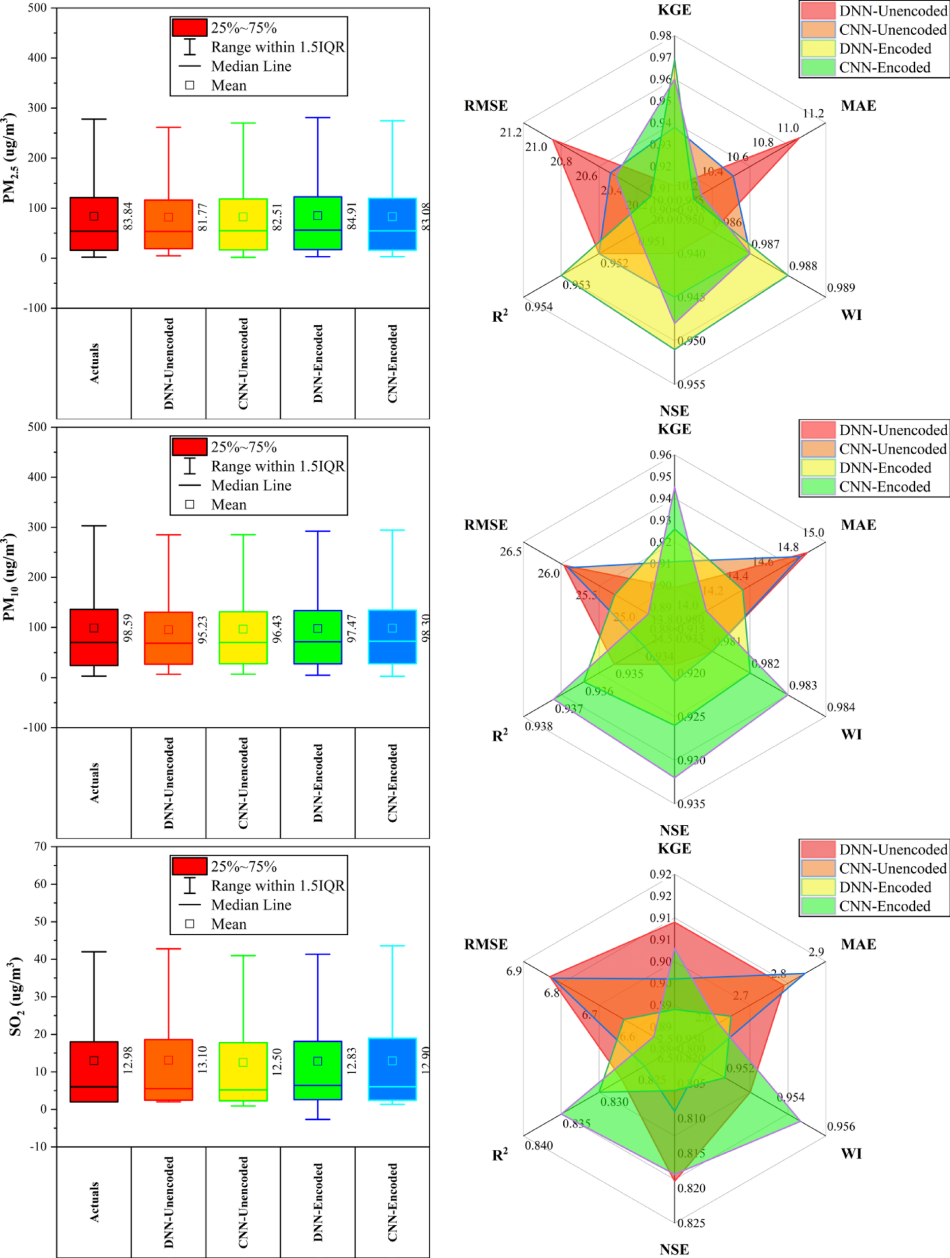
Forecasting results of six air pollutants using Unencoded and Encoded deep learning models at Shunyi Station.

At Shunyi Station (Fig. 9), CNN-Encoded models outperformed in ~ 60% of pollutants, achieving top metrics for CO (KGE: 0.946, R^2^: 0.907), PM_10_ (KGE: 0.945, R^2^: 0.937), and SO_2_ (MAE: 2.62 µg/m^3^, RMSE: 6.56 µg/m^3^). DNN-Encoded led in NO_2_ (KGE: 0.928, R^2^: 0.908), O_3_ (KGE: 0.938), and PM_2.5_ (KGE: 0.969, R^2^: 0.953). CO and O_3_ had the strongest model performance (KGE > 0.94), linked to high meteorological sensitivity. Dew point and month_cosine were key drivers in > 80% of cases, while rainfall had a < 10% impact. Overall, CNN-Encoded models provided superior forecasts when pollutant levels were strongly meteorology-dependent.

### Tiantan station

**Fig. 10:**
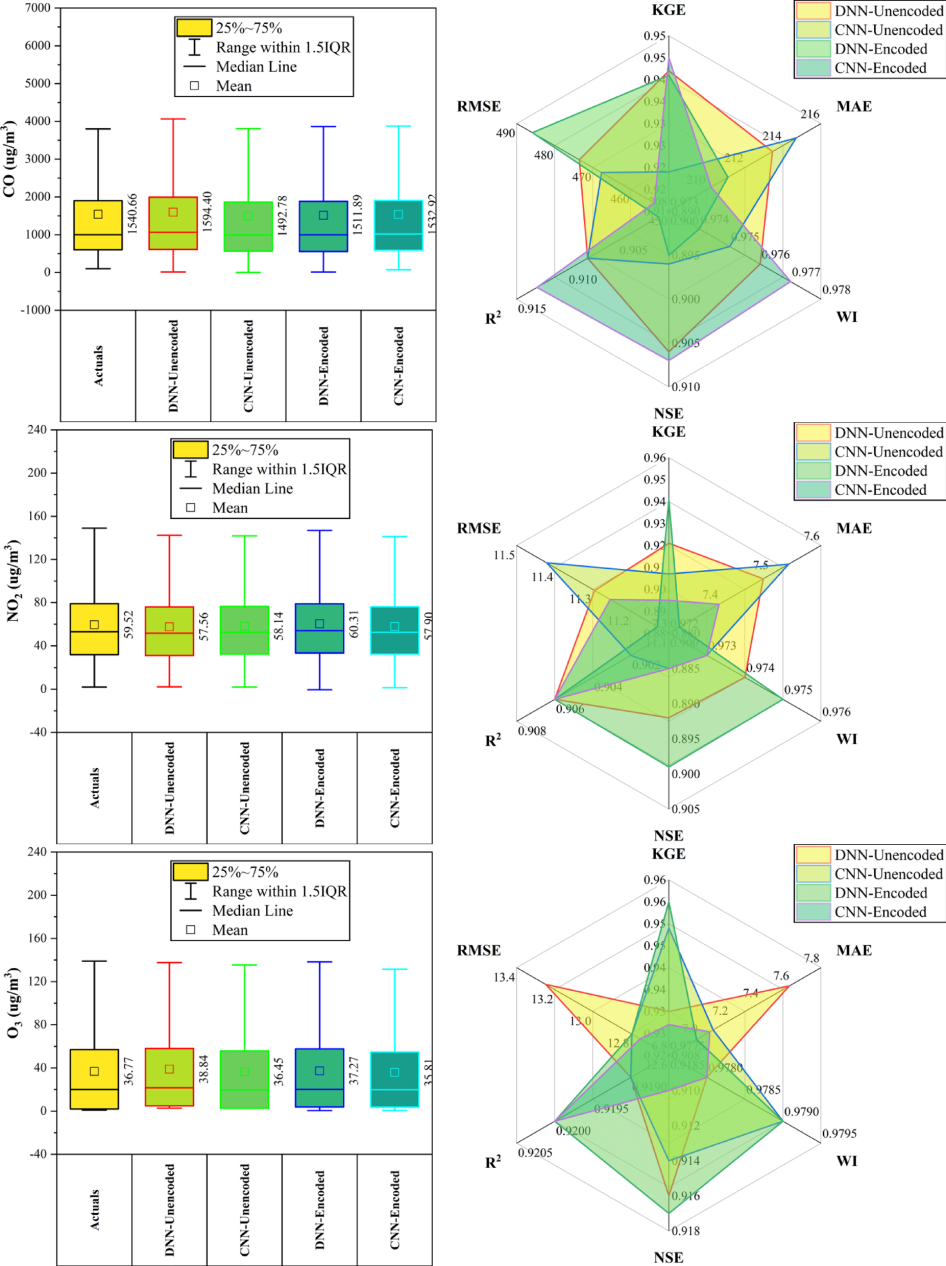

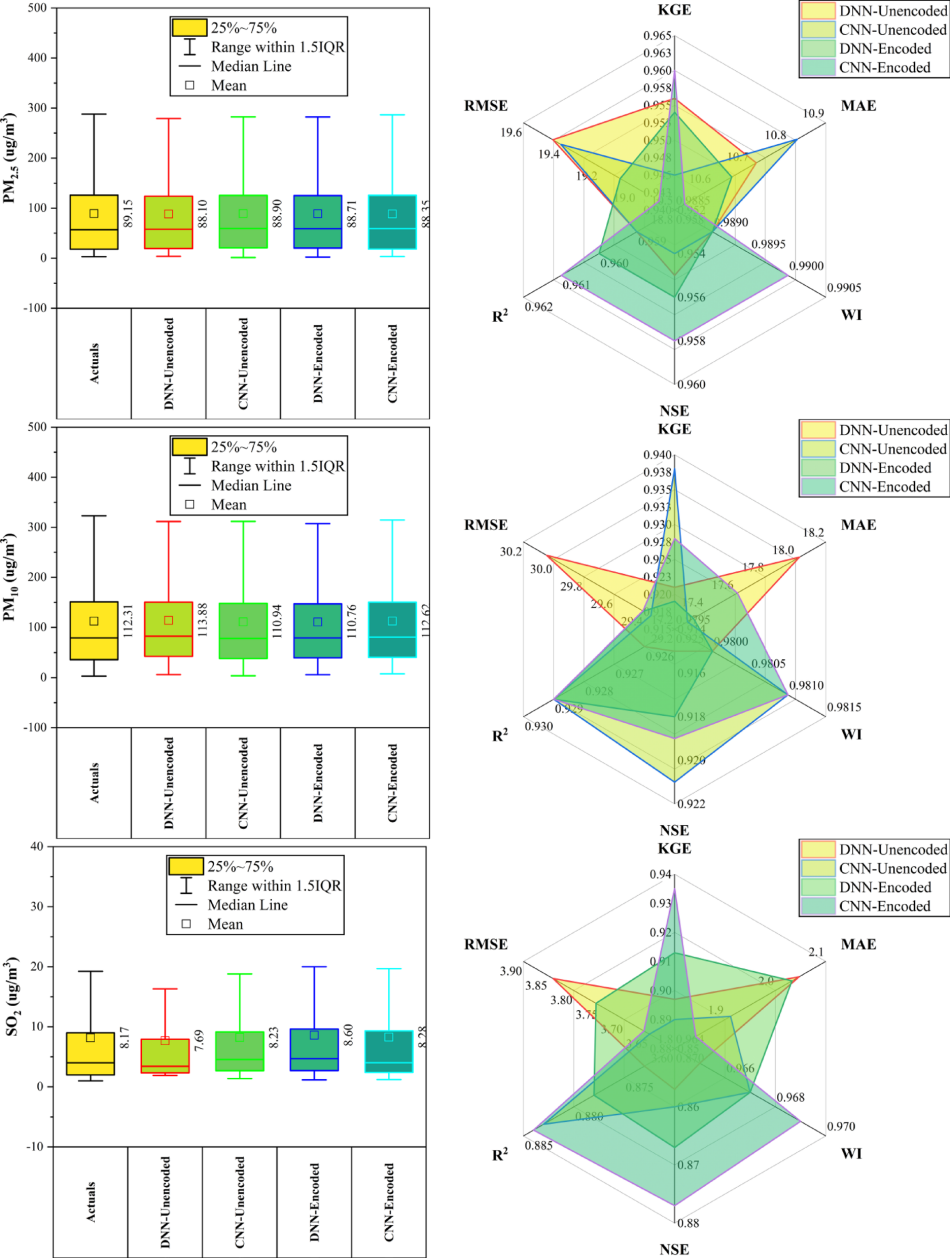
Forecasting results of six air pollutants using Unencoded and Encoded deep learning models at Tiantan Station.

At Tiantan Station (Fig. 10), encoded models outperformed unencoded ones in ~ 70% of cases. CNN-Encoded achieved top results for CO, PM_2.5_, and SO_2_ (KGE > 0.93, R^2^ > 0.88, MAE: 210.2 µg/m^3^ for CO; 10.53 µg/m^3^ for PM_2.5_). DNN-Encoded led in NO_2_ (KGE: 0.941, MAE: 7.32 µg/m^3^) and O_3_ (NSE: 0.917, R^2^: 0.92). PM_10_ was best predicted by CNN-Unencoded (KGE: 0.938). Key drivers (dew point, temperature, pressure, wind) influenced forecasts in > 80% of cases, while rainfall had a < 10% impact. Overall, encoded models improved accuracy by effectively capturing seasonal and atmospheric patterns.

### Wanliu station

**Fig. 11:**
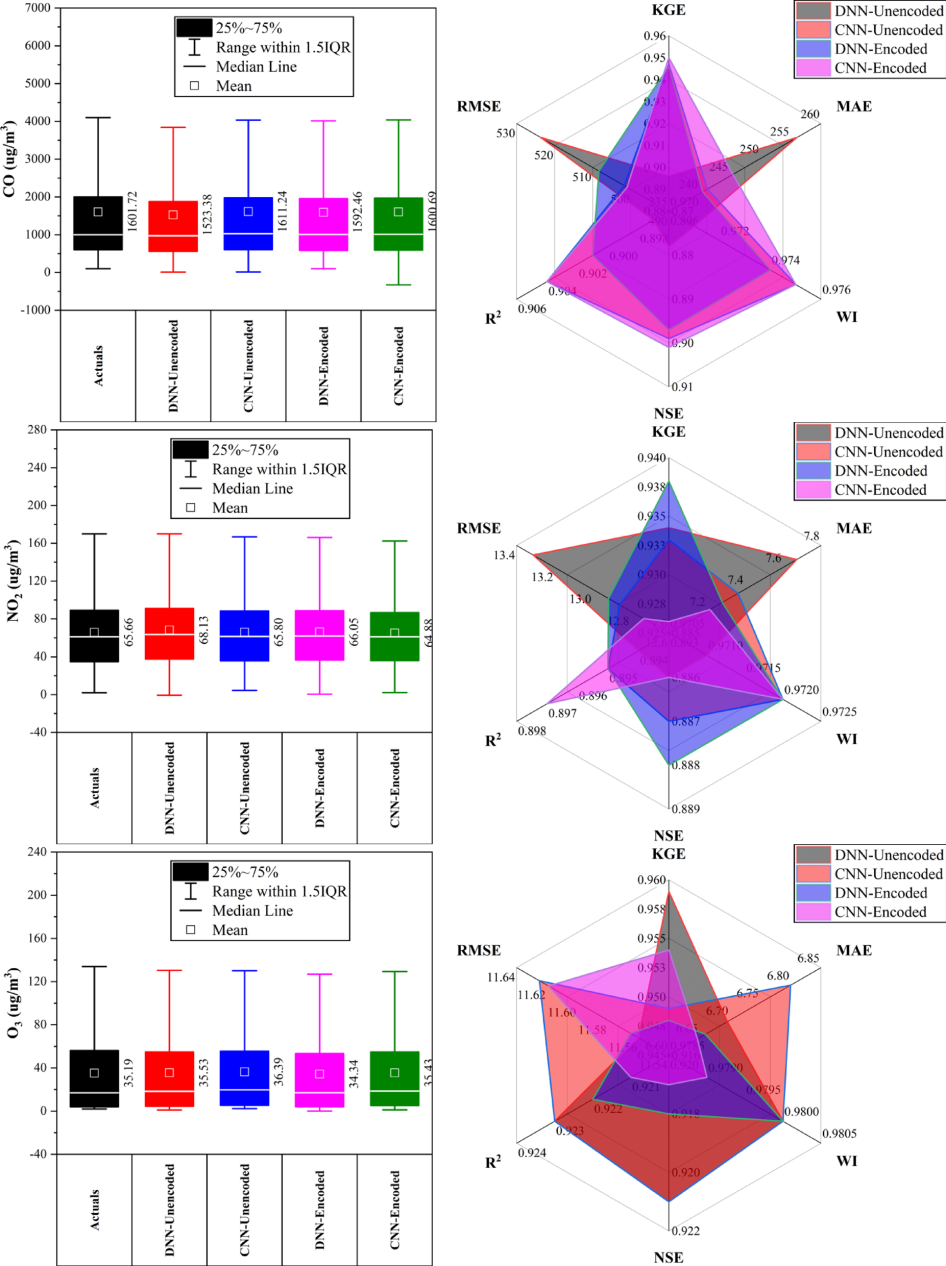

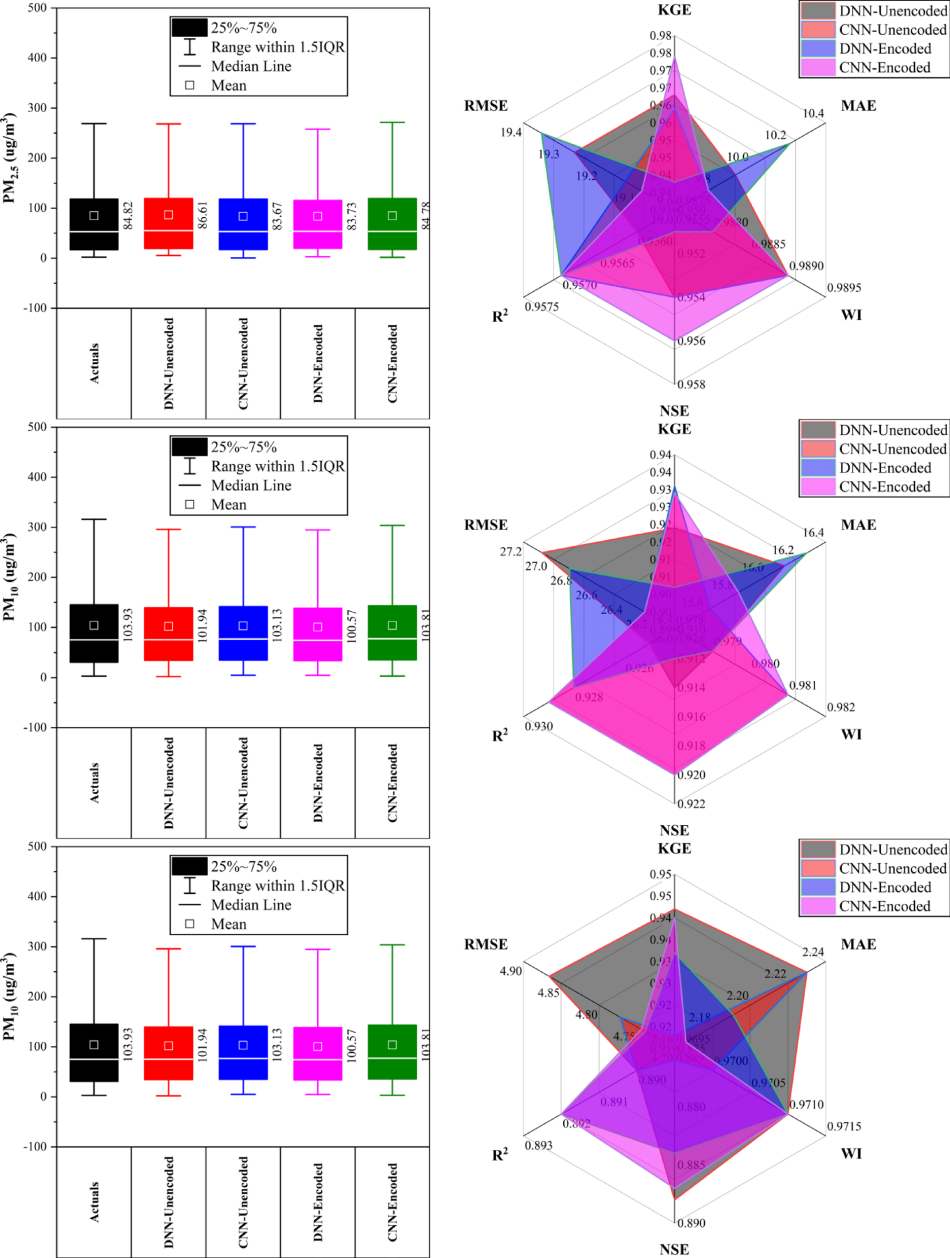
Forecasting results of six air pollutants using Unencoded and Encoded deep learning models at Wanliu Station.

At Wanliu Station (Fig. 11), CNN-Encoded models outperformed unencoded ones in ~ 75% of cases, achieving top accuracy for CO, PM_2.5_, and SO_2_ (KGE > 0.93, R^2^ > 0.88, MAE: 240.55 μg/m^3^ for CO; 9.78 μg/m^3^ for PM_2.5_). DNN-Encoded led NO_2_ forecasting (KGE: 0.938, MAE: 7.22 μg/m^3^) and O_3_ (NSE: 0.912, R^2^: 0.923). PM_10_ predictions were best by CNN-Unencoded (KGE: 0.931, MAE: 15.64 μg/m^3^). Key meteorological drivers (dew point, temperature, pressure, wind) influenced > 80% of results, while rainfall had minimal (< 10%) impact. Encoded features improved forecast accuracy by effectively capturing complex temporal and atmospheric patterns.

### Wanshouxigong station

**Fig. 12:**
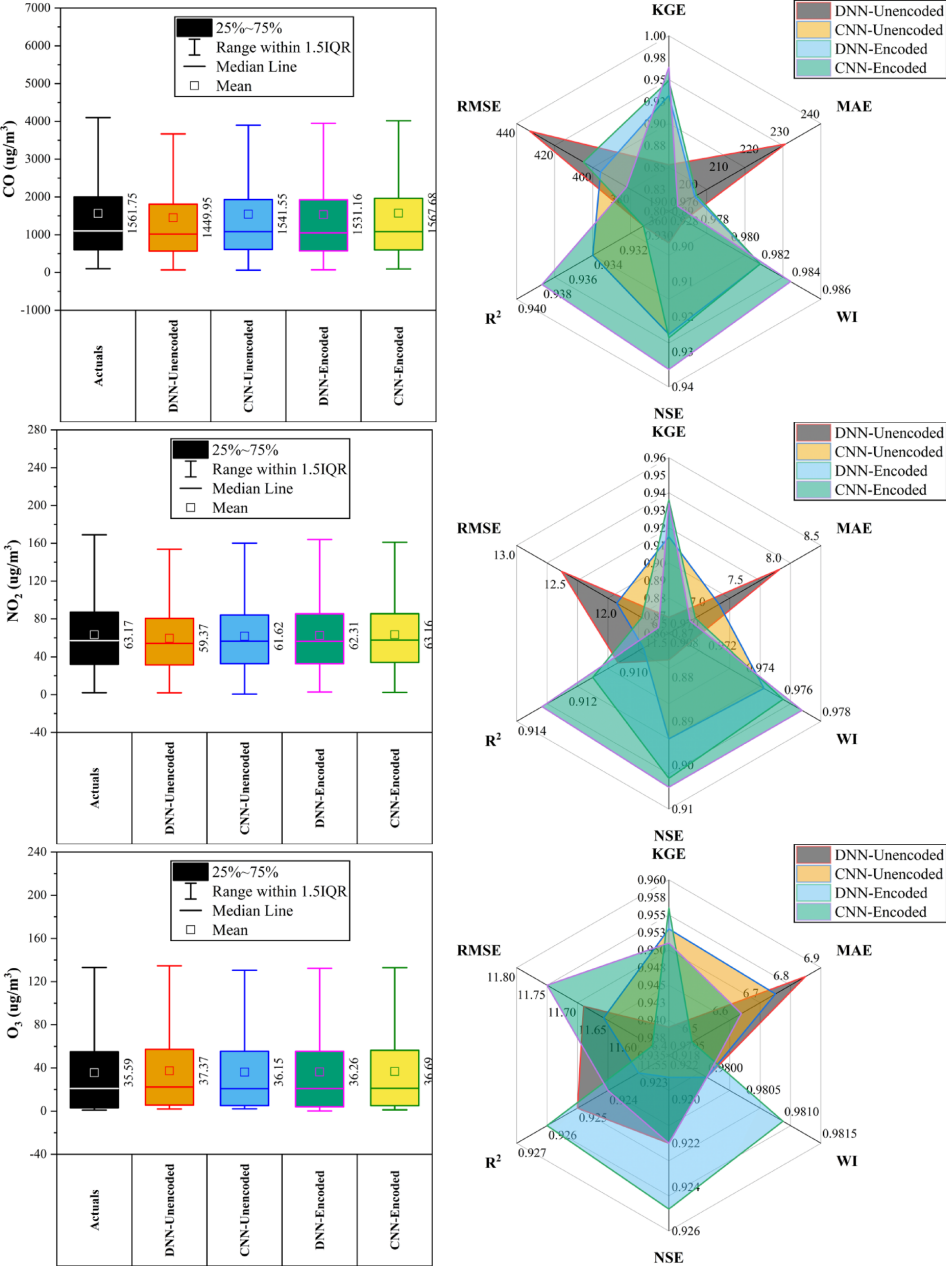

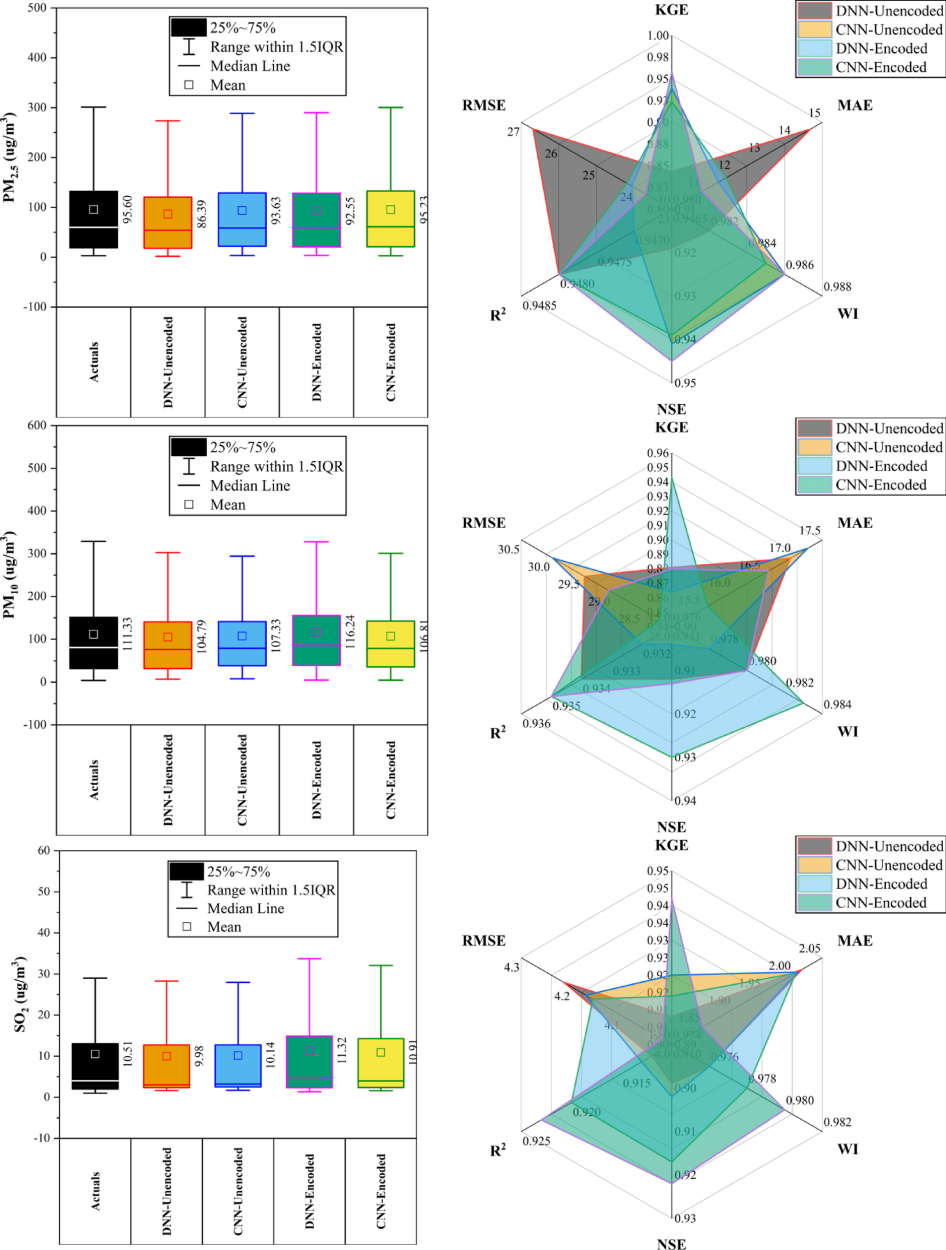
Forecasting results of six air pollutants using Unencoded and Encoded deep learning models at Wanshouxigong Station.

At Wanshouxigong Station (Fig. 12), CNN-Encoded models led in ~ 80% of cases, achieving the highest accuracy for CO (KGE: 0.964, R^2^: 0.938, MAE: 192.96 µg/m^3^) and PM_2.5_ (KGE: 0.958, R^2^: 0.948, MAE: 1.85 µg/m^3^). O_3_ was driven by temperature, wind, and hourly cycles; NO_2_ and SO_2_ showed strong seasonal (month) effects. PM_10_ performed best with DNN-Encoded (R^2^: 0.935, RMSE: 28.20 µg/m^3^). SO_2_ had lower accuracy but CNN-Encoded still improved errors (RMSE: 4.02 µg/m^3^). Rainfall had a minimal (< 10%) impact. Encoding boosted forecast reliability by capturing seasonal and short-term variations and reducing errors.

## Remarks and comparison

As per Tables 3, 4, 5, 6, 7, 8. The model performance across pollutants and locations is generally high, with R^2^ values mostly exceeding 0.85, reflecting strong predictive accuracy. PM_2.5_ and PM_10_ exhibit the highest and most consistent R^2^ scores, often above 0.94, indicating excellent model fit across all sites and methods. O_3_ predictions also show robust results, generally above 0.89. CO and NO_2_ show slightly more variation but still maintain strong performance, with CNN models—especially those using encoded inputs—tend to have a slight edge over DNNs. SO_2_ predictions are the most variable and generally lower, with some locations like Changping and Guanyuan showing R^2^ values closer to 0.76–0.78, suggesting more complexity or noise in the data. Locations such as Nongzhanguan, Wanshouxigong, and Tiantan consistently yield higher R^2^ values across pollutants and models, indicating more stable data or better model generalization, whereas Changping and Guanyuan often show comparatively lower performance. Overall, CNN architectures with encoded inputs generally offer marginal improvements, particularly for more challenging pollutants like SO_2_ and CO. Moreover, based on Table 9, CNN achieves the highest R^2^ in 70% of the cases (14 out of 20 combinations) across both unencoded and encoded features. DNN follows, ranking highest in 25% of cases, while LSTM leads only once (5%). ANN consistently underperforms, with the lowest R^2^ in 90% of the stations when features are encoded. Top-performing stations like Nongzhanguan, Tiantan, and Wanliu record R^2^ values above 0.96 with CNN, while lower-performing stations like Changping and Huairou have values around 0.94 or below, highlighting site-specific variability in model accuracy.

**Table 3:** Comparison of R^2^ values for pollutant CO across locations.

	DNN-Unencoded	CNN-Unencoded	DNN-Encoded	CNN-Encoded
Aotizhongxin	0.895	0.902	0.893	0.9
Changping	0.847	0.845	0.847	0.849
Dongsi	0.879	0.883	0.88	0.881
Guanyuan	0.881	0.904	0.904	0.9
Huairou	0.907	0.907	0.906	0.908
Nongzhanguan	0.923	0.923	0.921	0.926
Shunyi	0.907	0.904	0.9	0.905
Tiantan	0.908	0.908	0.901	0.913
Wanliu	0.897	0.904	0.901	0.904
Wanshouxigong	0.93	0.934	0.93	0.938

**Table 4:** Comparison of R^2^ values for pollutant NO_2_ across locations.

	DNN-Unencoded	CNN-Unencoded	DNN-Encoded	CNN-Encoded
Aotizhongxin	0.891	0.887	0.89	0.889
Changping	0.87	0.869	0.874	0.876
Dongsi	0.909	0.908	0.909	0.912
Guanyuan	0.916	0.913	0.915	0.914
Huairou	0.863	0.868	0.869	0.872
Nongzhanguan	0.917	0.917	0.919	0.919
Shunyi	0.904	0.902	0.908	0.905
Tiantan	0.906	0.902	0.906	0.906
Wanliu	0.894	0.895	0.895	0.897
Wanshouxigong	0.91	0.909	0.911	0.913

**Table 5:** Comparison of R^2^ values for pollutant O_3_ across locations.

	DNN-Unencoded	CNN-Unencoded	DNN-Encoded	CNN-Encoded
Aotizhongxin	0.932	0.932	0.934	0.932
Changping	0.904	0.907	0.908	0.908
Dongsi	0.908	0.909	0.907	0.909
Guanyuan	0.89	0.882	0.887	0.887
Huairou	0.921	0.926	0.923	0.928
Nongzhanguan	0.927	0.927	0.93	0.929
Shunyi	0.898	0.896	0.896	0.897
Tiantan	0.919	0.919	0.92	0.92
Wanliu	0.923	0.923	0.922	0.921
Wanshouxigong	0.925	0.923	0.926	0.924

**Table 6:** Comparison of R^2^ values for pollutant PM_2.5_ across locations.

	DNN-Unencoded	CNN-Unencoded	DNN-Encoded	CNN-Encoded
Aotizhongxin	0.9579	0.9579	0.956	0.956
Changping	0.9425	0.9427	0.9425	0.943
Dongsi	0.9518	0.9534	0.9541	0.9531
Guanyuan	0.955	0.9559	0.956	0.956
Huairou	0.9397	0.9401	0.9394	0.9408
Nongzhanguan	0.9614	0.9608	0.9617	0.9612
Shunyi	0.9517	0.9518	0.9526	0.9511
Tiantan	0.9586	0.9592	0.9602	0.9608
Wanliu	0.9565	0.957	0.9569	0.957
Wanshouxigong	0.9477	0.9475	0.948	0.9483

**Table 7:** Comparison of R^2^ values for pollutant PM_10_ across locations.

	DNN-Unencoded	CNN-Unencoded	DNN-Encoded	CNN-Encoded
Aotizhongxin	0.927	0.933	0.932	0.933
Changping	0.907	0.912	0.911	0.912
Dongsi	0.937	0.94	0.938	0.939
Guanyuan	0.93	0.933	0.933	0.933
Huairou	0.912	0.912	0.916	0.911
Nongzhanguan	0.932	0.933	0.933	0.933
Shunyi	0.935	0.934	0.936	0.937
Tiantan	0.926	0.929	0.929	0.929
Wanliu	0.925	0.929	0.928	0.929
Wanshouxigong	0.934	0.932	0.935	0.935

**Table 8:** Comparison of R^2^ values for pollutant SO_2_ across locations.

	DNN-Unencoded	CNN-Unencoded	DNN-Encoded	CNN-Encoded
Aotizhongxin	0.918	0.924	0.924	0.923
Changping	0.765	0.783	0.773	0.776
Dongsi	0.863	0.853	0.854	0.857
Guanyuan	0.777	0.772	0.765	0.782
Huairou	0.854	0.861	0.864	0.864
Nongzhanguan	0.861	0.878	0.861	0.88
Shunyi	0.827	0.824	0.83	0.835
Tiantan	0.873	0.883	0.878	0.884
Wanliu	0.89	0.89	0.892	0.892
Wanshouxigong	0.912	0.913	0.92	0.923

**Table 9:** Comparison of R^2^ values for PM_2.5_ prediction across stations using deep learning models with unencoded and encoded features.

	Unencoded features				Encoded features			
	DNN	ANN	CNN	LSTM	DNN	ANN	CNN	LSTM
Aotizhongxin	0.948	0.869	0.948	0.937	0.948	0.866	0.948	0.940
Changping	0.943	0.860	0.943	0.920	0.943	0.817	0.943	0.933
Dongsi	0.952	0.880	0.953	0.938	0.954	0.806	0.953	0.939
Guanyuan	0.955	0.935	0.956	0.944	0.956	0.841	0.956	0.947
Huairou	0.940	0.889	0.940	0.940	0.939	0.806	0.941	0.933
Nongzhanguan	0.961	0.783	0.961	0.944	0.962	0.828	0.961	0.928
Shunyi	0.952	0.867	0.952	0.936	0.953	0.819	0.951	0.938
Tiantan	0.959	0.878	0.959	0.942	0.960	0.850	0.961	0.944
Wanliu	0.957	0.893	0.957	0.940	0.957	0.826	0.957	0.947
Wanshouxigong	0.948	0.869	0.948	0.937	0.948	0.866	0.948	0.940

## Conclusion, limitations and future directions

This research highlights how effective deep learning models—specifically DNN and CNN frameworks—are at predicting major urban air pollutants across several monitoring locations using four years of hourly data. Both models delivered strong predictive performance, showing a high level of alignment between real and predicted pollutant values. Notably, the inclusion of feature encoding greatly boosted model accuracy, leading to steady gains of about 2–5% in key evaluation metrics like R^2^, NSE, and KGE.

The findings show that CNNs excelled at detecting spatial and temporal pollution patterns, particularly for pollutants such as CO and PM_2.5_. Meanwhile, DNNs demonstrated strong results across a wider range of pollutants. Feature encoding proved essential in enhancing the models’ ability to generalize and reduce prediction errors, underscoring the value of preprocessing in forecasting air quality over time.

Differences between monitoring sites showed the models could adapt to varying pollution trends and levels, reinforcing their usefulness in a variety of urban contexts. These insights suggest that deep learning, especially when supported by encoded features, holds significant potential for delivering accurate and scalable air quality predictions—tools that could be crucial for city planning and public health efforts.

Despite these strong results, the study is not without limitations. The deep learning models require significant computational resources—GPU or cloud-based infrastructure—which may limit their application in resource-constrained settings. Moreover, external environmental drivers such as meteorological data (temperature, wind speed, humidity), traffic emissions, and industrial output were not included in the modeling pipeline. Incorporating these factors could potentially improve model accuracy by up to 10–15%, based on evidence from other related literature. Additionally, the geographic scope of the study was limited to Beijing, reducing the model’s generalizability to regions with different climatic and socio-economic profiles.

The practical implications of this research are remarkable. High-accuracy pollutant forecasting, with R^2^ values above 0.90 in many cases, can support early warning systems, enabling city authorities to issue timely health advisories and reduce exposure risks. The integration of these models into smart city infrastructure could lead to more efficient urban planning, including dynamic traffic control and targeted industrial regulation. Furthermore, the framework demonstrated in this study provides a scalable foundation for AI-driven air quality management, capable of being deployed in various urban areas.

For future research, increasing the input feature space to include meteorological and socioeconomic variables is essential. Preliminary studies indicate that adding weather-related variables can increase forecasting accuracy by 8–12%. Model interpretation should also be prioritized using tools such as SHAP or attention mechanisms to uncover the influence of specific features on predictions. Furthermore, incorporating data from multiple cities with different pollution profiles would enhance its adaptability and general applicability. Finally, transitioning to real-time, cloud-based deployment can provide scalable, on-demand predictions. Hybrid models that combine deep learning with physical or statistical modeling may improve prediction robustness by 10–20%, offering a promising direction for next-generation environmental forecasting systems.

## Data Availability

All data reported in the manuscripts are available from the corresponding author upon justified request.
